# Immunogenicity and Safety of Heterologous *Versus* Homologous Prime-Boost Regimens With BBIBP-CorV and Ad26.COV2.S COVID-19 Vaccines: A Multicentric, Randomized, Observer-Blinded Non-inferiority Trial in Madagascar and Mozambique

**DOI:** 10.1093/cid/ciaf130

**Published:** 2025-07-22

**Authors:** Patrícia Ramgi, Mohamadou Siribie, Njariharinjakamampionona Rakotozandrindrainy, Odete Bule, Harshvardhan Shrivastava, Lígia Chambule, Eun Lyeong Park, Carina Fernando, Jéssica Boque, Rezelda Macuiana, Ravomialisoa Razafimanantsoa, Ndrainaharimira Rakotozandrindrainy, Tsiriniaina J L Razafindrabe, Antenaina N Rakotoarisoa, Tiana M Raminosoa, Herinirina L Derandrainy, Masinirina M Rakotoson, Cynthia S S de Silva, Mirna Mutombene, Carmélia Massinga, José P Langa, Tobin Guarnacci, Sophie S Y Kang, Sue Kyoung Jo, Hyon Jin Jeon, Jean-Louis Excler, Yunkai Yang, Shiyu Wang, Jonathan D Sugimoto, Jae Seung Yang, Byoung-Shik Shim, Tabea Binger, Igor U Capitine, Asma B Aziz, Ju Yeon Park, Deok Ryun Kim, Raphaël Rakotozandrindrainy, Ilesh V Jani, Birkneh Tilahun Tadesse, Florian Marks

**Affiliations:** Instituto Nacional de Saúde, Maputo, Mozambique; International Vaccine Institute, Seoul, Republic of Korea; Burkina Institute of Global Health, Ouagadougou, Burkina Faso; Madagascar Institute for Vaccine Research, University of Antananarivo, Antananarivo, Madagascar; Instituto Nacional de Saúde, Maputo, Mozambique; International Vaccine Institute, Seoul, Republic of Korea; Instituto Nacional de Saúde, Maputo, Mozambique; International Vaccine Institute, Seoul, Republic of Korea; Instituto Nacional de Saúde, Maputo, Mozambique; Instituto Nacional de Saúde, Maputo, Mozambique; Instituto Nacional de Saúde, Maputo, Mozambique; Madagascar Institute for Vaccine Research, University of Antananarivo, Antananarivo, Madagascar; Madagascar Institute for Vaccine Research, University of Antananarivo, Antananarivo, Madagascar; Madagascar Institute for Vaccine Research, University of Antananarivo, Antananarivo, Madagascar; Madagascar Institute for Vaccine Research, University of Antananarivo, Antananarivo, Madagascar; Madagascar Institute for Vaccine Research, University of Antananarivo, Antananarivo, Madagascar; Madagascar Institute for Vaccine Research, University of Antananarivo, Antananarivo, Madagascar; Madagascar Institute for Vaccine Research, University of Antananarivo, Antananarivo, Madagascar; Instituto Nacional de Saúde, Maputo, Mozambique; Instituto Nacional de Saúde, Maputo, Mozambique; Instituto Nacional de Saúde, Maputo, Mozambique; Instituto Nacional de Saúde, Maputo, Mozambique; International Vaccine Institute, Seoul, Republic of Korea; International Vaccine Institute, Seoul, Republic of Korea; International Vaccine Institute, Seoul, Republic of Korea; International Vaccine Institute, Seoul, Republic of Korea; Madagascar Institute for Vaccine Research, University of Antananarivo, Antananarivo, Madagascar; Department of Medicine, Cambridge Institute of Therapeutic Immunology and Infectious Disease, University of Cambridge, Cambridge, United Kingdom; International Vaccine Institute, Seoul, Republic of Korea; China National Biotec Group Company Limited, China; China National Biotec Group Company Limited, China; International Vaccine Institute, Seoul, Republic of Korea; Department of Epidemiology, University of Washington, Seattle, Washington, USA; International Vaccine Institute, Seoul, Republic of Korea; International Vaccine Institute, Seoul, Republic of Korea; International Vaccine Institute, Seoul, Republic of Korea; Instituto Nacional de Saúde, Maputo, Mozambique; International Vaccine Institute, Seoul, Republic of Korea; International Vaccine Institute, Seoul, Republic of Korea; International Vaccine Institute, Seoul, Republic of Korea; Madagascar Institute for Vaccine Research, University of Antananarivo, Antananarivo, Madagascar; Instituto Nacional de Saúde, Maputo, Mozambique; International Vaccine Institute, Seoul, Republic of Korea; Division of Clinical Pharmacology, Department of Laboratory Medicine, Karolinska Institutet, Karolinska University Hospital Huddinge, Stockholm, Sweden; Center for Innovative Drug Development and Therapeutic Trials for Africa, College of Health Sciences, Addis Ababa University, Addis Ababa, Ethiopia; International Vaccine Institute, Seoul, Republic of Korea; Madagascar Institute for Vaccine Research, University of Antananarivo, Antananarivo, Madagascar; Department of Medicine, Cambridge Institute of Therapeutic Immunology and Infectious Disease, University of Cambridge, Cambridge, United Kingdom; Heidelberg Institute of Global Health, University of Heidelberg, Heidelberg, Germany; The Hong Kong Jockey Club Global Health Institute, Hong Kong Special Administrative Region, China

**Keywords:** SARS-CoV-2, BBIBP-CorV, Ad26.COV2.S, heterologous, homologous

## Abstract

**Background:**

Data on immunogenicity and safety of heterologous prime-boost (HePB) regimens using the BBIBP-CorV and Ad26.COV2.S have not yet been reported in sub-Saharan Africa.

**Methods:**

We conducted a randomized, observer-blinded, non-inferiority trial assessing the immunogenicity and safety of HePB regimens using BBIBP-CorV and Ad26.COV2.S, in adults aged 18–65 years. Participants enrolled, were stratified by baseline severe acute respiratory syndrome coronavirus 2 (SARS-CoV-2) serostatus, and randomized into four arms in a 1:1:1:1 ratio: A1 (BBIBP-CorV, Ad26.COV2.S), A2 (BBIBP-CorV, BBIBP-CorV), B1 (Ad26.COV2.S, BBIBP-CorV), and B2 (placebo, Ad26.COV2.S), administered at 28-day intervals. Fifteen participants in each arm were randomized separately in the immunology subset at a ratio of 1:1:1:1. Primary endpoints were the geometric mean titers (GMTs) of anti–SARS-CoV-2 neutralizing antibodies (nAbs) against SARS-CoV-2 Omicron variant BA.1 and safety at 4 weeks after second vaccination. The non-inferiority margin was 0.67 fold difference in geometric mean ratio (GMR) between the ratio of GMTs in the heterologous *versus* corresponding homologous arms.

**Results:**

A total of 369 participants were randomized, and 367 of them received at least one dose of vaccine. Participants were between 18 and 65 years of age. Four weeks after second dose, GMT of nAbs in arms A1 and A2 was 802.7 (95% confidence interval [CI]: 635.3–1014.3) and 202.6 (95% CI: 150.8–272.1), respectively, with an adjusted GMR of 4.2 (2-sided 95% CI: 2.9–5.9). GMTs were 603.6 (95% CI: 446.1–816.7) and 725.7 (95% CI: 539.5–976.1) in arms B1 and B2, respectively, with an adjusted GMR of 0.8 (2-sided 95% CI: .5–1.2). Three serious adverse events were reported and none of them were related to the vaccination.

**Conclusions:**

The noninferiority criterion was met only in arm A1 *versus* A2. HePB regimens were safe and well tolerated.

**Clinical Trials Registration:**

NCT04998240.

## Teaser

Only heterologous regimen, using BBIBP-CorV vaccine as primary dose and Ad26.COV2.S vaccine as booster dose, induced robust humoral response 4 weeks after the booster as compared to the corresponding homologous regimen. Both heterologous and homologous regimens were safe.

Coronavirus disease 2019 (COVID-19), caused by the severe acute respiratory syndrome coronavirus 2 (SARS-CoV-2), was first detected in Wuhan, China, in December 2019 [1]. With the rapid spread of the virus, the World Health Organization (WHO) declared a pandemic on 11 March 2020 [2]. This pandemic has been among the most serious global health threats in recent history and has caused major health, economic, social, and educational disruptions worldwide [3–5]. This pandemic triggered an unprecedented race to develop safe, effective vaccines for global use and to limit the pandemic's devastating consequences, through extensive collaborative and coordinating efforts among scientific communities, industries, governments, nongovernmental organizations, and donors [6].

In the first year after the pandemic's onset, the vaccines developed and authorized for emergency use were administered as two doses with a homologous prime-boost (HoPB) regimen, with the same vaccine type administered for both the primary and booster doses, except for the Janssen vaccine, which was administered as a single dose [6]. Subsequently, interest grew in the heterologous boost COVID-19 vaccine strategy, to elicit a greater, more durable, and broader putative immune response against emerging virulent strains, and to meet vaccine demands by overcoming vaccine shortages during mass vaccination campaigns [7]. Studies in the past decade have indicated that heterologous prime-boost (HePB) immunizations are feasible and can be more immunogenic than HoPB immunizations. The heterologous boost approach combines the immunological properties of multiple vaccines and may promote maturation of antibody affinity and influence the breadth of immunization through the use of different antigens, vectors, delivery routes, doses, or adjuvants at different times [8–11]. Although many studies have assessed the immunogenicity and reactogenicity of heterologous boost COVID-19 vaccination, most were conducted outside of sub-Saharan Africa (SSA) [7, 12, 13]. Therefore, a critical need existed to generate data in SSA to optimize vaccine uptake [14, 15].

To expand COVID-19 vaccine use in SSA, we assessed whether HePB regimens with the inactivated BBIBP-CorV Sinopharm vaccine and modified adenovirus vector Ad26.COV2.S Janssen vaccine might be safe and induce an immune response against the SARS-CoV-2 Omicron variant BA.1 at least comparable to that induced by equivalent HoPB regimens in Malagasy and Mozambican adults. These two vaccines were widely used in mass vaccination programs in SSA early in the pandemic [16].

## METHODS

### Ethics and Regulatory Statement

The trial was approved by the Comite Nacional de Bioteica Para Saúde (CNBS) – Mozambique; Autoridade Nacional Reguladora de Medicamentos (ANARME) – Mozambique; Comité d´Ethique de la Recherche Biomédicale (CERBM) – Madagascar; and the International Vaccine Institute Institutional Review Board. The study complied with the Declaration of Helsinki, the Council for International Organizations of Medical Sciences International Ethical guidelines, and the Good Clinical Practice guidelines of the International Council for Harmonisation of Technical Requirements for Pharmaceuticals for Human Use. The study was registered at ClinicalTrials.gov (identifier NCT04998240).

### Trial Design and Participants

We conducted a phase 2 multicenter, observer-blinded, randomized, noninferiority trial to assess the immunogenicity and safety of HePB regimens at four weeks after the booster dose with BBIBP-CorV and Ad26.COV2.S vaccines. Recruitment was conducted across three sites in Mozambique (Centro de Investigação e Treino da Polana Caniço, Universidade Eduardo Mondlane, and Xipamanine Health Care Facility in Maputo) and one site in Madagascar (Madagascar Institute for Vaccine Research in Antananarivo). Trial participants were enrolled and randomized to the following arms (with prime and boost regimens indicated in parentheses) in a 1:1:1:1 ratio: arm A1 (BBIBP-CorV, Ad26.COV2.S), arm A2 (BBIBP-CorV, BBIBP-CorV), arm B1 (Ad26.COV2.S, BBIBP-CorV) and arm B2 (placebo, Ad26.COV2.S). Fifteen participants in each arm were randomized separately in an immunology subset at a ratio 1:1:1:1. A placebo was administered first in arm B2 to respect the one-dose recommendation for the emergency use authorization for the Ad26.COV2.S study, while maintaining the blinding of observers and participants to regimen assignment.

Adults 18–65 years of age, with no or well-controlled mild to moderate comorbidities, who were living in Maputo City and the Maputo province area in Mozambique, or in Antananarivo city and Antananarivo province area in Madagascar, were invited to participate. The key exclusion criteria were known infection with human immunodeficiency virus and/or hepatitis B virus; any clinically significant abnormal findings, as judged by the research physician; prior COVID-19 vaccination; confirmed active SARS-CoV-2 infection at enrollment; history of bleeding disorders; continuous anticoagulant use; use of immunoglobulins or/and any blood products in the 3 months before COVID-19 vaccination; history of allergy to vaccine components and/or excipients; and breastfeeding, pregnancy, or intent to conceive during the study period.

### Trial Procedures

Participants were recruited from the general community in the study catchment areas. The trial teams held local meetings within the community to explain COVID-19, the need for COVID-19 vaccination, the purpose of the trial, the trial's general procedures, eligibility criteria and informed consent procedures, and potential benefits and risks of vaccination, by using advertising materials approved by the ethics committees. After these meetings, a list of volunteers interested in participating was compiled, and volunteers were invited to trial sites for consent and screening visit. Eligible participants were enrolled and randomized to receive two intramuscular injections of the assigned study product four weeks apart.

A computer-generated randomization list was prepared by an independent biostatistician with no involvement in study implementation. The list contained sequential numbers unique to each participant, and block randomization was used to ensure balance among treatment arms, as well as to prevent potential unmasking. Two randomization lists—one with randomization number only and the other with randomization number and vaccine allocation—were prepared. The former was shared only with blinded trial staff and was used for enrolling and assigning randomization numbers to participants, whereas the latter was shared with the unblinded study team (pharmacists and vaccine administrators).

Vaccines were administered by well-trained trial nurses. Apart from the designated study site personnel responsible for vaccine administration and vaccine accountability, the study investigators and all participants were blinded to vaccine allocation until the database was locked before statistical analysis.

### Immunogenicity Assessment

The primary immunogenicity endpoints were assessed from serum samples obtained at baseline (before the first vaccination); 4 weeks after the first vaccination and before the second vaccination; and 4 weeks after the second vaccination. The level of anti–SARS-CoV-2 neutralizing antibody (nAb) titers against the Omicron variant BA.1 was assessed with SARS-CoV-2–specific plaque reduction neutralization test (PRNT). PRNT titers were expressed as 50% nAb titers (PRNT_50_).

### Safety Assessments

All vaccinees were observed at the study sites for at least 30 minutes after each vaccination for immediate reactogenicity assessment, then followed up for safety assessment at specific time points. Local and systemic solicited adverse events (AEs) were recorded on a diary card within 7 and 14 days, respectively, after each vaccination, and were reviewed by the research physicians 14 days after each dose. Unsolicited AEs were recorded until 28 days after each vaccination. Serious adverse events (SAEs) and adverse events of special interest (AESIs) were recorded throughout the study from the first dose.

### Outcomes

The primary immunogenicity outcome was the geometric mean titers (GMTs) of anti–SARS-CoV-2 nAbs against the Omicron variant BA.1 4 weeks after the second vaccination in the heterologous *versus* homologous arms, regardless of the SARS-CoV-2 infection serostatus at baseline. The geometric mean ratio (GMR) and geometric mean fold rise (GMFR) for nAbs to SARS-CoV-2 Omicron variant BA.1 were assessed. The safety endpoints were the incidence of SAEs, AESIs from the first vaccination until four weeks after the second vaccination, local solicited AEs (seven days after each vaccination), systemic solicited AEs (14 days after each vaccination), and unsolicited AEs including changes in the laboratory parameters four weeks after each vaccination.

### Statistical Analysis

The sample size was calculated according to the following assumptions: a non-inferiority margin of 0.67 fold difference in GMR between the ratio of GMTs in the heterologous *versus* homologous arms, a coefficient of variation in GMTs on the original scale of 1.12 based on the data available from BBIBP-CorV phase 1/2 publication [17], a true ratio of GMTs on the original scale of 1, and a drop-out rate of 10%. Therefore, a total sample size of 360 participants, 90 per treatment arm, was determined to be required to achieve 80% power at a 1-sided 2.5% significance level, and to detect the non-inferiority of immunogenicity of the heterologous arms (A1 and B1) *versus* their corresponding homologous arms (A2 and B2).

Immunogenicity analysis was conducted with the *per protocol* set including all eligible randomized participants who received all planned administrations, had at least one immunogenicity (nAb) test 28 days after the booster dose, and had no important protocol deviations. The GMTs and 95% confidence intervals (CIs) for each treatment arm were calculated. HePB regimens were considered non-inferior to HoPB regimens if the lower limit of the two-sided 95% CI of the GMR between the heterologous boost arm and the corresponding homologous arm exceeded 0.67 (WHO Technical Report Series). Immunogenicity titers that were below the lower limit of quantification (LLOQ), which is 10, was set to half the LLOQ in the analysis.

The GMR, GMFR, and associated 95% CI were also calculated in each treatment arm. The GMR of GMTs and GMFR and associated 95% CI were calculated as the antilogarithm of the difference and its 95% CI between the mean of the log-transformed data in heterologous arm *versus* homologous arm, after adjustment for age, baseline SARS-CoV-2 serostatus, and cohort in a generalized linear regression model. The safety endpoints were summarized descriptively. AEs are reported as frequencies and percentages for each arm, according to severity, seriousness, and relatedness with vaccination. Safety analysis was conducted for all randomized participants who received at least 1 dose of the study products.

No multiplicity adjustment of the comparison was performed.

## RESULTS

### Participant Disposition, Baseline Demographics, and SARS-CoV-2 Antibody Serostatus Characteristics

**Figure 1.**
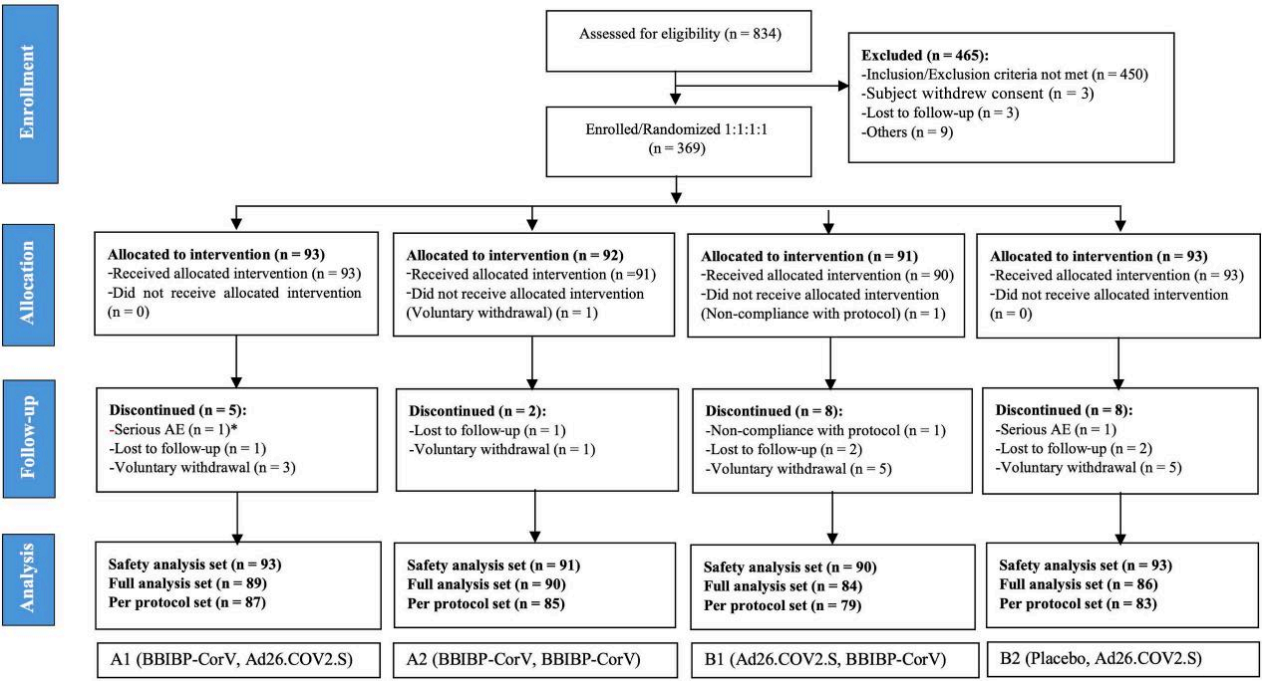
Trial profile showing the disposition of the study participants to each of the 4 study arms.

The CONSORT flow diagram is presented in Figure 1. Participants were recruited from 19 December 2021 to 26 September 2022, during the BA.1 Omicron outbreak, in Madagascar and Mozambique. Of 834 participants screened, 369 met the eligibility criteria and were randomized to 1 of 4 treatment arms (A1, A2, B1, and B2). The participants' baseline characteristics, including demographic characteristics and baseline SARS-CoV-2 antibody serostatus, were well balanced between the heterologous and corresponding homologous study arms (Table 1).

**Table 1.** Baseline Demographics and Serostatus to SARS-CoV-2 Antibodies of the Study Participants, Based on Safety Analysis Set

Parameter	Arm A1	Arm A2	Arm B1	Arm B2
Total No. of participants	93	91	90	93
Sex, No. (%)				
Male	61 (65.6)	57 (62.6)	61 (67.8)	67 (72.0)
Female	32 (34.4)	34 (37.4)	29 (32.2)	26 (28.0)
Ethnicity, No. (%)				
Black	52 (55.9)	50 (54.9)	48 (53.3)	52 (55.9)
Other (Malagasy)	41 (44.1)	41 (45.1)	42 (46.7)	41 (44.1)
Age, y				
Mean (SD)	29.0 (9.1)	27.5 (7.9)	27.3 (7.6)	26.3 (7.0)
Median	26.0	25.0	25.0	24.0
Min, Max	18.0, 60.0	18.0, 49.0	18.0, 51.0	18.0, 50.0
Height, cm				
Mean (SD)	164.5 (9.7)	166.1 (10.7)	165.6 (9.7)	166.8 (10.3)
Median	163.5	165.3	165.9	167.2
Min, Max	145.3, 187.0	144.5, 192.0	144.0, 185.6	135.7, 188.0
Weight, kg				
Mean (SD)	58.2 (9.6)	58.7 (10.7)	59.7 (11.8)	58.4 (9.5)
Median	58.0	57.2	58.5	57.5
Min, Max	35.3, 87.6	37.9, 99.0	40.6, 111.1	35.8, 76.9
BMI, kg/m^2^				
Mean (SD)	21.5 (3.2)	21.3 (3.6)	21.7 (3.9)	21.0 (2.8)
Median	20.6	20.4	21.0	20.5
Min, Max	16.0, 34.6	16.1, 37.6	16.8, 41.8	16.6, 31.5
Serostatus to SARS-CoV-2 antibodies, No. (%)				
Seropositive	78 (83.9)	77 (84.6)	78 (86.7)	78 (83.9)
Seronegative	15 (16.1)	14 (15.4)	12 (13.3)	15 (16.1)

Study arms are as follows: A1, BBIBP-CorV (Sinopharm vaccine), Ad26.COV2.S (Janssen vaccine); A2, BBIBP-CorV, BBIBP-CorV; B1, Ad26.COV2.S, BBIBP-CorV; B2, placebo, Ad26.COV2.S.

Abbreviations: BMI, body mass index; Max, maximum value; Min, minimum value; SARS-CoV-2, severe acute respiratory syndrome coronavirus 2; SD, standard deviation.

### Immunogenicity

Among participants primed with BBIBP-CorV vaccine, the GMT of nAbs to SARS-CoV-2 Omicron variant BA.1, four weeks after second dose, was 202.6 (95% CI: 150.8–272.1) in participants receiving the homologous booster (arm A2) and 802.7 (95% CI: 635.3–1014.3) in those receiving the heterologous booster (arm A1). The adjusted GMR four weeks after the second vaccination was 4.2 (two-sided 95% CI: 2.9–5.9). The lower limit of the two-sided 95% CI of the GMR was greater than the margin of 0.67, thus, indicating the non-inferiority of arm A1 to arm A2. Arm A1 was statistically superior to arm A2 in inducing nAbs to SARS-CoV-2 Omicron variant BA.1. The GMFR was comparable between participants primed with BBIBP-CorV and boosted with Ad26.COV2.S (A1) and those boosted with BBIBP-CorV (A2) four weeks after the prime dose. A four-fold increase of GMTs in arm A1 was observed four weeks after the booster dose as compared to the baseline, whereas it remained unchanged in arm A2 (Table 2).

**Table 2.** Geometric Mean Titers, Geometric Mean Fold Rises, and Geometric Mean Ratio of Neutralizing Antibodies Against SARS-CoV-2 Omicron Variant BA.1, Based on *Per Protocol* Set

Parameter	Time Point	Arm A1 (n = 87 Participants)		Arm A2 (n = 85 Participants)		Arm B1 (n = 79 Participants)		Arm B2 (n = 83 Participants)	
		Value (95% CI)	SD	Value (95% CI)	SD	Value (95% CI)	SD	Value (95% CI)	SD
GMT	Baseline	182.9 (134.1–249.4)	4.3	148.2 (101.4–216.6)	5.8	157.7 (108.2–229.7)	5.4	188.4 (129.1–274.9)	5.7
	4 weeks	253.0 (187.6–341.1)	4.1	198.6 (149.3–264.2)	3.8	574.2 (418.9–787.0)	4.1	165.5 (113.4–241.6)	5.7
	8 weeks	802.7 (635.3–1014.3)	3.0	202.6 (150.8–272.1)	3.9	603.6 (446.1–816.7)	3.9	725.7 (539.5–976.1)	3.9
GMFR	4 weeks	1.4 (1.1–1.7)	2.5	1.3 (1.1–1.7)	3.9	3.6 (2.7–4.9)	3.6	0.9 (.8–1.0)	2.0
	8 weeks	4.4 (3.4–5.6)	3.2	1.4 (1.0–1.8)	3.9	3.8 (2.9–5.0)	3.3	4.4 (3.4–5.7)^a^	3.2
GMR	Time point	Crude ratio (A1/A2)		Adjusted ratio^b^ (A1/A2)		Crude ratio (B1/B2)		Adjusted ratio^b^ (B1/B2)	
	4 weeks	1.3 (.8–1.9)		1.3 (.9–1.9)		3.5 (2.1–5.7)^c^		3.4 (2.1–5.3)^c^	
	8 weeks	3.9 (2.7–5.8)		4.2 (2.9–5.9)		0.8 (.6–1.3)		0.8 (.5–1.2)	

Study arms are as follows: A1, BBIBP-CorV (Sinopharm vaccine), Ad26.COV2.S (Janssen vaccine); A2, BBIBP-CorV, BBIBP-CorV; B1, Ad26.COV2.S, BBIBP-CorV; B2, placebo, Ad26.COV2.S.

Abbreviations: CI: confidence interval; GMFR, geometric mean fold rise; GMR, geometric mean ratio (geometric mean titer for heterologous/homologous); GMT, geometric mean titer; SD, geometric standard deviation for log-transformed (base 2) titer for the geometric mean titer and geometric standard deviation for log-transformed (base 2) fold rise titer from baseline for the geometric mean fold rise.

^a^GMFR value is calculated from week 4, not week 0, for B2 (placebo, Ad26.COV2.S).

^b^The adjusted GMR and 95% CI are derived using the generalized linear regression model by adjusting for age, baseline SARS-CoV-2 serostatus, and cohort (general/immunology subset) as randomization design variable.

^c^GMR comparison between Ad26.COV2.S vs placebo.

Among participants primed with Ad26.COV2.S vaccine and placebo, the GMTs of the nAbs to SARS-CoV-2 Omicron variant BA.1, four weeks after second dose, were 725.7 (95% CI: 539.5–976.1) in the homologous booster arm (arm B2) and 603.6 (95% CI: 446.1–816.7) in the heterologous booster arm (arm B1). The adjusted GMR 4 weeks after the second vaccination was 0.8 (two-sided 95% CI: .5–1.2). The lower limit of the two-sided 95% CI of the GMR was less than the margin of 0.67. Thus, the non-inferiority criterion was not met in arm B1 *versus* arm B2. Participants primed with Ad26.COV2.S elicited a significantly higher GMFR 4 weeks after the prime dose than participants in the homologous arm (arm B2). However, the GMFR was not statistically different between these arms four weeks after the booster dose (Table 2).

In exploratory subgroup analysis, the GMTs for nAbs to SARS-CoV-2 Omicron variant BA.1 induced four weeks after the second dose in participants with SARS-CoV-2 antibody seropositivity at baseline were 905.5 (95% CI: 733.5–1117.8) in arm A1 and 242.0 (95% CI: 181.0–323.5) in arm A2. Among participants with SARS-CoV-2 antibody seronegativity at baseline, the GMTs for nAbs to SARS-CoV-2 Omicron variant BA.1 induced four weeks after the second dose in arm A1 and arm A2 were 450.3 (95% CI: 175.6–1154.7) and 75.7 (95% CI: 27.4–209.4), respectively. The GMTs were significantly higher in participants only with SARS-CoV-2 antibody seropositivity at baseline in arm A1 *versus* arm A2. However, no statistically significant difference was observed in the GMTs for nAbs to SARS-CoV-2 Omicron variant BA.1 induced four weeks after the booster dose in participants with SARS-CoV-2 seropositivity and seronegativity in arm B1 *versus* arm B2 (Table 3). Moreover, when stratified by baseline serostatus, homologous and heterologous boost using BBIBP-CorV did not significantly elicit the GMTs of nAbs against SARS-CoV-2 Omicron variant BA.1, while the HePB using the Ad26.COV2.S elicited significant GMTs of nAbs against SARS-CoV-2 Omicron variant BA.1 (Table 3).

**Table 3.** Geometric Mean Titers of Neutralizing Antibodies Against SARS-CoV-2 Omicron Variant BA.1, According to Baseline Serostatus, Based on *Per Protocol* Set

Serostatus	Time Point	Arm A1		Arm A2		Arm B1		Arm B2	
		No.^a^	GMT (95% CI)	No.^a^	GMT (95% CI)	No.^a^	GMT (95% CI)	No.^a^	GMT (95% CI)
Seropositive	Baseline	72	240.5 (175.6–329.5)	72	203.2 (139.5–295.9)	68	215.8 (149.8–311.0)	72	265.5 (186.2–378.6)
	4 weeks	72	320.6 (235.1–437.3)	72	230.6 (174.6–304.5)	68	702.0 (510.1–966.0)	72	223.4 (155.3–321.2)
	8 weeks	72	905.5 (733.5–1117.8)	72	242.0 (181.0–323.5)	68	722.7 (530.5–984.7)	72	863.2 (661.9–1125.6)
Seronegative	Baseline	15	49.1 (24.0–100.5)	13	25.8 (9.6–69.1)	11	22.6 (8.8–58.2)	11	19.9 (7.7–51.4)
	4 weeks	15	81.1 (40.5–162.5)	13	86.9 (30.1–250.7)	11	165.8 (66.9–410.8)	11	23.3 (7.7–70.8)
	8 weeks	15	450.3 (175.6–1154.7)	13	75.7 (27.4–209.4)	11	198.2 (83.0–473.6)	11	233.2 (56.8–956.9)

Study arms are as follows: A1, BBIBP-CorV (Sinopharm vaccine), Ad26.COV2.S (Janssen vaccine); A2, BBIBP-CorV, BBIBP-CorV; B1, Ad26.COV2.S, BBIBP-CorV; B2, placebo, Ad26.COV2.S.

Abbreviations: CI: confidence interval; GMT, geometric mean titer.

^a^No. of participants.

### Safety

Three SAEs were reported during this trial and were deemed unrelated to the vaccination: Two due to induced abortions (one in each of arms A1 and B1), and one in arm B2 due to mental and behavioral disorders associated with psychoactive substance use, which led to the hospitalization of the participant. No AESIs were reported.

**Figure 2.**
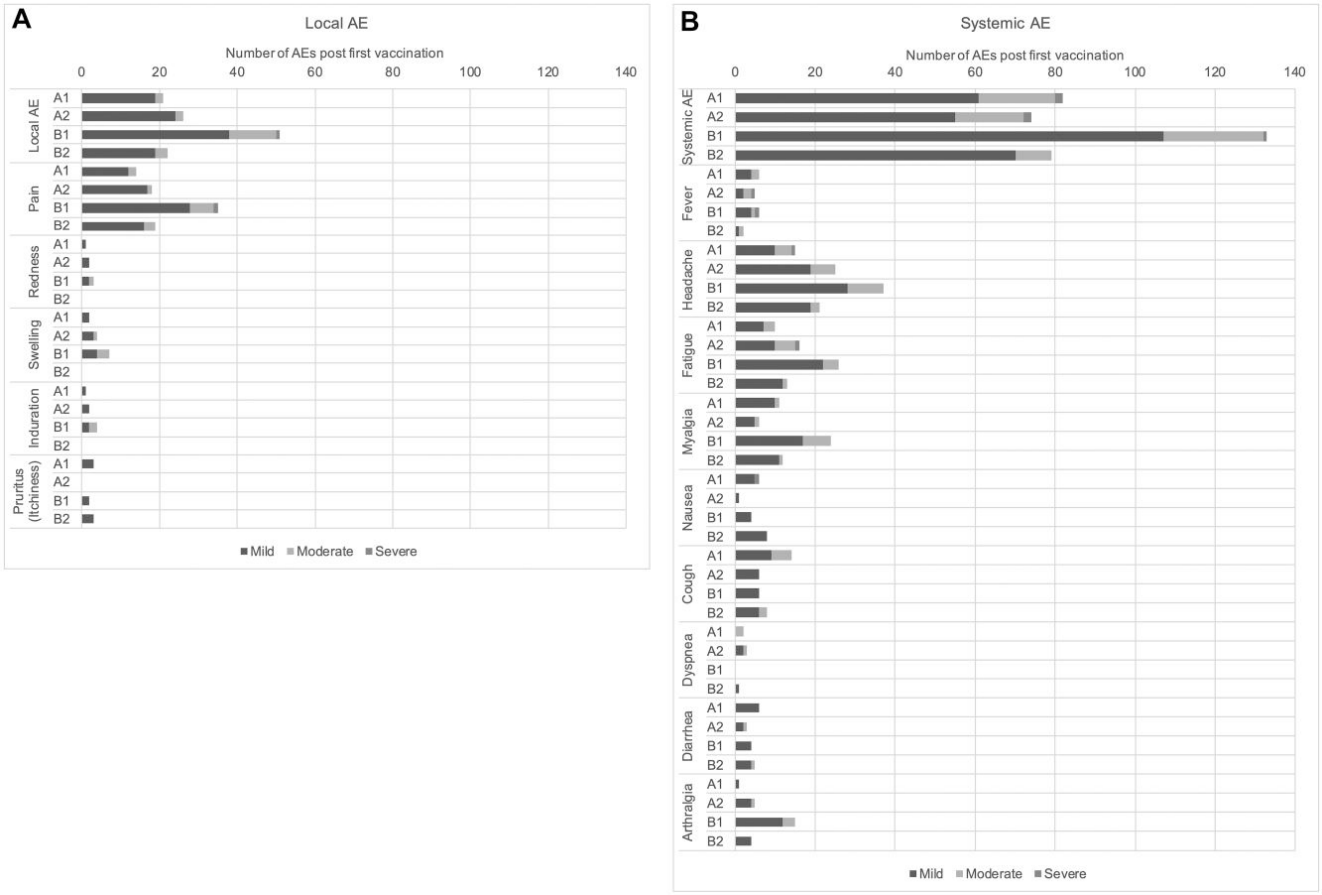
Solicited local and common systemic reactions that occurred respectively within 7 days and 14 days **of first vaccination**, by severity and study arm. Abbreviation: AE, adverse event.

**Figure 3.**
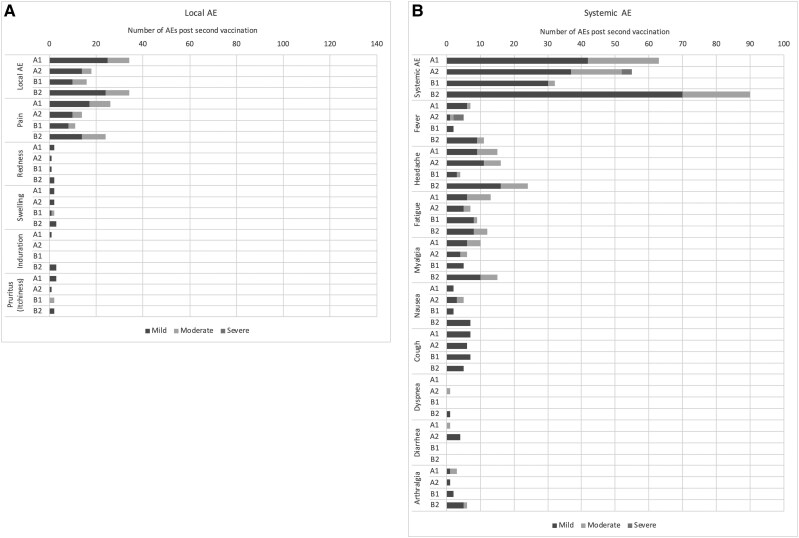
Solicited local and common systemic reactions that occurred respectively within 7 days and 14 days **of second vaccination**, by severity and study arm. Abbreviation: AE, adverse event.

The incidence of local solicited AEs within 7 days after any vaccination in each of the study arms is presented in Table 4. Most of these events were mild to moderate and only 1 (pain at the injection site) was severe in arm B1. All of these AEs were judged to be associated with the vaccination (Table 4, Supplementary Tables 4 and 5). The most frequent local solicited AEs were injection site pain after both first and second vaccinations. All AEs were transient (Figures 2 and 3 and Supplementary Tables 4 and 5).

**Table 4.** Summary of Adverse Events Four Weeks After Any Vaccination

Adverse Event	A1 (n = 93)		A2 (n = 91)		B1 (n = 90)		B2 (n = 93)	
	No. of Events	No. (%)^a^	No. of Events	No. (%)^a^	No. of Events	No. (%)^a^	No. of Events	No. (%)^a^
Local solicited AEs	55	38 (40.9)	44	28 (30.8)	67	36 (40.0)	56	29 (31.2)
Severity								
Mild	44	32 (34.4)	38	24 (26.4)	48	34 (37.8)	43	23 (24.7)
Moderate	11	11 (11.8)	6	6 (6.6)	18	9 (10.0)	13	12 (12.9)
Severe	0	0 (0.0)	0	0 (0.0)	1	1 (1.1)	0	0 (0.0)
Relatedness^b^								
Not related	0	0 (0.0)	0	0 (0.0)	0	0 (0.0)	0	0 (0.0)
Related	55	38 (40.9)	44	28 (30.8)	67	36 (40.0)	56	29 (31.2)
Systemic solicited AEs	145	36 (38.7)	129	39 (42.9)	165	41 (45.6)	169	40 (43.0)
Severity								
Mild	103	32 (34.4)	92	28 (30.8)	137	39 (43.3)	140	38 (40.9)
Moderate	40	16 (17.2)	32	19 (20.9)	27	14 (15.6)	29	14 (15.1)
Severe	2	2 (2.2)	5	2 (2.2)	1	1 (1.1)	0	0 (0.0)
Relatedness^b^								
Not related	0	0 (0.0)	0	0 (0.0)	0	0 (0.0)	5	2 (2.2)
Related	145	36 (38.7)	129	39 (42.9)	165	41 (45.6)	164	40 (43.0)
Unsolicited AEs	7	5 (5.4)	11	6 (6.6)	4	3 (3.3)	4	3 (3.2)
Severity								
Mild	5	3 (3.2)	4	4 (4.4)	2	2 (2.2)	3	2 (2.2)
Moderate	2	2 (2.2)	5	3 (3.3)	2	1 (1.1)	1	1 (1.1)
Severe	0	0 (0.0)	2	1 (1.1)	0	0 (0.0)	0	0 (0.0)
Relatedness^b^								
Not related	4	3 (3.2)	9	5 (5.5)	2	2 (2.2)	2	2 (2.2)
Related	3	2 (2.2)	2	2 (2.2)	2	2 (2.2)	2	1 (1.1)
Serious AE	1	1 (1.1)	0	0 (0.0)	1	1 (1.1)	1	1 (1.1)
AE of special interest	0	0 (0.0)	0	0 (0.0)	0	0 (0.0)	0	0 (0.0)

Data are presented as No. of participants who reported events unless otherwise indicated.

Study arms are as follows: A1, BBIBP-CorV (Sinopharm vaccine), Ad26.COV2.S (Janssen vaccine); A2, BBIBP-CorV, BBIBP-CorV; B1, Ad26.COV2.S, BBIBP-CorV; B2, placebo, Ad26.COV2.S.

Abbreviation: AE, adverse event.

^a^No. (%) of participants who reported events.

^b^Related: possibly, probably, or definitely related; not related: unlikely or not related.

The incidence of systemic solicited AEs within 14 days after any vaccination in each of the study arms was presented in Table 4. These events were transient and most of them were mild to moderate, except 2 cases (one case of headache and one case of nausea) in arm A1, 5 cases (four cases of fever and one case of fatigue) in arm A2, and 1 case of fever in arm B1 (Table 4, Supplementary Tables 4 and 5). These AEs were all assessed as associated with the vaccination except five in arm B2 (Table 4, Supplementary Tables 4 and 5). The most common systemic solicited AEs in all treatment arms after both the first and second vaccinations were headache, fatigue, myalgia, cough, and fever (Figures 2 and 3 and Supplementary Tables 4 and 5).

The incidences of unsolicited AEs until 4 weeks after any vaccination in arms A1, A2, B1, and B2 were summarized (Table 4). Two of them were assessed as severe and due to elevated liver enzymes (Supplementary Tables 6 and 7).

## DISCUSSION

BBIBP-CorV and Ad26.COV2.S vaccines were widely available in early stages in SSA in the rollout of vaccines against COVID-19 [16]. Four weeks after the booster dose, a robust humoral response was elicited against the Omicron variant BA.1 only in participants primed with BBIBP-CorV vaccine and boosted with Ad26.COV2.S vaccine, as compared with those who received two doses BBIBP-CorV vaccine. In addition, the HePB regimens, as compared with the HoPB regimens, were safe and well tolerated.

To our knowledge, this study is the first to assess heterologous boost strategies using these two vaccines in SSA. A randomized, controlled, observer-blinded trial of HePB immunization with the inactivated CoronaVac vaccine and recombinant adenovirus type 5–vectored vaccine Convidecia in healthy adults 18–59 years of age in China has also demonstrated that the HePB regimen with Convidecia after priming with CoronaVac (either two dose or one dose) induced higher GMTs of nAbs against the live SARS-CoV-2 than homologous boost with CoronaVac at 14 and 28 days after the booster dose [18]. In addition, a prospective serological cohort study in Thailand has indicated that boosting with the adenovirus-vectored vaccine ChAdOx1-S after priming with CoronaVac elicits a significantly better antibody response than two doses of CoronaVac [19]. Moreover, non-inferiority and quasi-experimental studies in Thailand have reported different results in terms of GMR of the anti–receptor binding domain antibody concentration to wild-type SARS-CoV-2, at four weeks after the booster dose, in the homologous *versus* heterologous arms receiving inactivated vaccines (CoronaVac, BBIBP-CorV) and ChAdOx1-S vaccine [20, 21]. Similarly, the nAbs concentrations elicited against the Omicron variant BA.1 in participants with SARS-CoV-2 antibody seropositivity were at least as high in arm A1 as in arm A2. The non–statistically significant difference of the GMTs of nAbs observed in participants with SARS-CoV-2 antibody seronegativity between arm A1 and arm A2 should be interpretated with caution due to the small sample sizes of participants in both arms. However, we observed no statistically significant difference between the nAbs elicited against the Omicron variant BA.1 in arm B1 *versus* arm B2, and according to baseline SARS-CoV-2 antibody serostatus. Moreover, the humoral responses against SARS-CoV-2 Omicron variant BA.1 induced at four weeks post–second dose in arms A1, B1, and B2 were similar, and the humoral responses against SARS-CoV-2 Omicron variant BA.1 induced four weeks after vaccination with BBIBP-CorV vaccine were similar to the humoral responses at baseline (arms A1 and A2). This underlined the limited ability of BBIBP-CorV vaccine to induce potent humoral response against the SARS-CoV-2 Omicron variant BA.1 as compared to Ad26.COV2.S vaccine.

Our findings also indicated similar safety profiles of the heterologous boost regimens compared with the homologous boost regimens four weeks after the booster dose. Acceptable safety profiles have also been reported with the homologous boost regimens with Ad26.COV2.S and BBIBP-CorV vaccines *versus* placebo [17, 22, 23]. Similarly, HePB schedules using BBIBP-CorV and Ad26.COV2.S vaccines, compared with homologous schedules, have also shown satisfactory safety profiles [18–21, 24, 25].

This study has several limitations. First, the overall drop-out rate was slightly high and ranged from 7% to 10%. Drop-out cases occurred mainly in Mozambique, where the recruitment started during the lockdown period. After lockdown restrictions were lifted, participants went searching for employment, often outside the study area, which resulted in a high rate of consent withdrawal due to their unavailability. Fortunately, the sample size remained powered to assess the primary immunogenicity endpoint. Moreover, we assessed immune responses against only the Omicron variant BA.1, which was associated with outbreaks in the southern part of SSA during the recruitment period. Extending the immunogenicity assessment to broad and major virus genotypes, including the wild-type strain and other SARS-CoV-2 variants of concern, would be more informative. Finally, the data reported here only informed about the immune response until four weeks after the booster dose. We are currently unable to determine whether higher concentrations of nAbs to the Omicron variant BA.1 measured 28 days after the booster dose in arm A1 might result in sustained persistence of vaccine-induced antibodies.

## CONCLUSIONS

Our study demonstrated that a heterologous regimen using BBIBP-CorV vaccine as a primary dose and Ad26.COV2.S vaccine as a booster dose induced a robust humoral response four weeks after the booster in adults as compared to the corresponding homologous arm. In addition, regardless of baseline serostatus, the HePB regimen using Ad26.COV2.S vaccine appeared to induce significant GMTs of nAbs against SARS-CoV-2 Omicron variant BA.1 whereas the GMTs elicited by HoPB and HePB regimens using BBIBP-CorV vaccine appeared non-significant. Moreover, our results seem to indicate that HePB regimens (arm A1 and arm B1) are likely to be at least as immunogenic as a single dose of Ad26.COV2.S (arm B2). Overall, our findings further support the flexibility of the heterologous boost strategy using these two vaccines.

## Supplementary Material

ciaf130_Supplementary_Data

## Supplementary Data

Supplementary materials are available at *Clinical Infectious Diseases* online. Consisting of data provided by the authors to benefit the reader, the posted materials are not copyedited and are the sole responsibility of the authors, so questions or comments should be addressed to the corresponding author.

## Notes

***Authors contributions*.** F. M., B. T. T., I. V. J., Rap. R., M. S., J. D. S., D. R. K., and J. Y. P. conceived and designed the study. I. V. J., Rav. R., P. R., I. U. C., and Nj. R. were responsible for data acquisition. D. R. K., E. L. P., and J. Y. P. analyzed the data. M. S., P. R., and Nj. R. wrote the first draft of the manuscript. All authors interpreted the data, reviewed the manuscript critically for important intellectual content, and approved the final draft of the manuscript.

***Acknowledgments*.** The authors express their deep gratitude to the participants in this study, the community health workers and field staff for their hard work, and the administration and health officials at both sites who generously provided facilities and support for the conduct of the study. They are grateful to Paul Oloo, Saurabh Ahuja, and Dorota Zdankiewicz from the Coalition for Epidemic Preparedness Innovations for oversight; and the data safety and monitoring board members (Dr Christine Manyando, Professor Taesung Park, Dr Shayhana Ganesh, Professor Tumani Corrah, and Dr Edson Luís Bernardo) for providing insightful comments and guidance.

***Disclaimer*.** The funding organization had no role in the design, collection, analysis, or interpretation of data, or in the writing of the manuscript. China National Biotec Group Company Limited had no role in the design, collection and analysis. The findings and conclusions in this report are those of the authors and do not necessarily represent the official position of the International Vaccine Institute, the Instituto Nacional de Saúde, the University of Antananarivo, the Madagascar Institute for Vaccine Research, or any of the collaborating partners in this study.

***Financial support.*** This work was funded by the Coalition for Epidemic Preparedness Innovations (CEPI) (grant number PRJ-6447).

***Supplement sponsorship*.** This article appears as part of the supplement “COVID-19 Vaccine Research in Africa,” sponsored by the International Vaccine Institute and funded by the Coalition for Epidemic Preparedness Innovations (CEPI).
