# Supplementary material for: Immunogenicity and Safety of Heterologous Versus Homologous Prime-Boost Regimens With BBIBP-CorV and Ad26.COV2.S COVID-19 Vaccines: A Multicentric, Randomized, Observer-Blinded Non-inferiority Trial in Madagascar and Mozambique
Source: Clin Infect Dis. 2025 Jul 22;80(Suppl 1):S37–46. doi: 10.1093/cid/ciaf130 (PMC12282515; doi:10.1093/cid/ciaf130)
Supplement: ciaf130_Supplementary_Data [file ciaf130_supplementary_data.docx]

**Supplementary Materials**

[**Supplementary Table 1.** Baseline demographic information and serostatus to SARS-CoV-2 antibodies of the study participants (Full Analysis Set) 2](#_Toc182539595)

[**Supplementary Table 2*.*** Baseline demographic information and serostatus to SARS-CoV-2 antibodies of the study participants (Per Protocol Set) 3](#_Toc182539596)

[**Supplementary Table 3.** Geometric Mean Titers, Geometric Mean-Fold Rises and Geometric Mean Ratio of the neutralizing antibodies against SARS-Cov-2 Omicron variant BA.1, (full analysis set). 4](#_Toc182539597)

[**Supplementary Table 4.** Local and Systemic solicited reactions occurred respectively within 7 days and 14 days post vaccination by study arms 5](#_Toc182539598)

[**Supplementary Table 5.** Local and Systemic solicited reactions occurred respectively within 7 days and 14 days post vaccination with severity by study arms 7](#_Toc182539599)

[**Supplementary Table 6.** Unsolicited adverse events (SOC/PT) occurred respectively within 28 days post vaccination by study arms 14](#_Toc182539600)

[**Supplementary Table 7.** Unsolicited adverse events occurred respectively within 28 days post vaccination with severity by study arms 17](#_Toc182539601)

**Supplementary Table 1.** Baseline demographic information and serostatus to SARS-CoV-2 antibodies of the study participants (Full Analysis Set)

| **Parameters** | **A1 Arm** | **A2 Arm** | **B1 Arm** | **B2 Arm** |
| --- | --- | --- | --- | --- |
| Participants, n | **89** | **90** | **84** | **86** |
| Sex, n (%) |  |  |  |  |
| Male | 59 (66.3%) | 57 (63.3%) | 57 (67.9%) | 62 (72.1%) |
| Female | 30 (33.7%) | 33 (36.7%) | 27 (32.1%) | 24 (27.9%) |
| Ethnicity, n (%) |  |  |  |  |
| Black | 48 (53.9%) | 49 (54.4%) | 44 (52.4%) | 46 (53.5%) |
| Other (Malagasy) | 41 (46.1%) | 41 (45.6%) | 40 (47.6%) | 40 (46.5%) |
| Age (years) |  |  |  |  |
| Mean (SD) | 29.2 (9.2) | 27.4 (7.8) | 27.5 (7.7) | 26.5 (7.0) |
| Median | 27.0 | 25.0 | 25.0 | 24.0 |
| Min, Max | 19.0, 60.0 | 18.0, 49.0 | 18.0, 51.0 | 18.0, 50.0 |
| Height (cm) |  |  |  |  |
| Mean (SD) | 164.5 (9.9) | 166.3 (10.5) | 165.2 (9.5) | 166.3 (10.4) |
| Median | 164.6 | 165.4 | 165.8 | 167.1 |
| Min, Max | 145.3, 187.0 | 144.5, 192.0 | 144.0, 183.0 | 135.7, 188.0 |
| Weight (kg) |  |  |  |  |
| Mean (SD) | 58.2 (9.8) | 58.7 (10.8) | 58.9 (11.0) | 58.0 (9.5) |
| Median | 58.0 | 57.5 | 58.3 | 57.2 |
| Min, Max | 35.3, 87.6 | 37.9, 99.0 | 40.6, 111.1 | 35.8, 76.9 |
| BMI (kg/m^2^) |  |  |  |  |
| Mean (SD) | 21.5 (3.3) | 21.3 (3.6) | 21.6 (3.7) | 21.0 (2.9) |
| Median | 20.5 | 20.4 | 20.9 | 20.3 |
| Min, Max | 16.0, 34.6 | 16.1, 37.6 | 16.8, 41.8 | 16.6, 31.5 |
| Serostatus to SARS-CoV2 Antibodies, n (%) |  |  |  |  |
| Seropositive | 74 (83.1%) | 76 (84.4%) | 72 (85.7%) | 74 (86.0%) |
| Seronegative | 15 (16.9%) | 14 (15.6%) | 12 (14.3%) | 12 (14.0%) |
| Abbreviations: A1: BBIBP-CorV, Ad26.COV2.S; A2: BBIBP-CorV, BBIBP-CorV; B1: Ad26.COV2.S, BBIBP-CorV; B2: Placebo, Ad26.COV2.S; N: number of total participants; n: number of participants in the category; %: percentages (100*n/N); SD: Standard Deviation; Min.: Minimum value; Max.: Maximum value; BMI: Body mass index. | | | | |

**Supplementary Table 2*.*** Baseline demographic information and serostatus to SARS-CoV-2 antibodies of the study participants (Per Protocol Set)

| **Parameters** | **A1 Arm** | **A2 Arm** | **B1 Arm** | **B2 Arm** |
| --- | --- | --- | --- | --- |
| Participants, n | **87** | **85** | **79** | **83** |
| Sex, n (%) |  |  |  |  |
| Male | 57 (65.5%) | 53 (62.4%) | 53 (67.1%) | 59 (71.1%) |
| Female | 30 (34.5%) | 32 (37.6%) | 26 (32.9%) | 24 (28.9%) |
| Ethnicity, n (%) |  |  |  |  |
| Black | 47 (54.0%) | 45 (52.9%) | 43 (54.4%) | 43 (51.8%) |
| Other (Malagasy) | 40 (46.0%) | 40 (47.1%) | 36 (45.6%) | 40 (48.2%) |
| Age (years) |  |  |  |  |
| Mean (SD) | 29.3 (9.2) | 27.3 (7.9) | 27.8 (7.8) | 26.5 (7.2) |
| Median | 27.0 | 25.0 | 26.0 | 24.0 |
| Min, Max | 19.0, 60.0 | 18.0, 49.0 | 18.0, 51.0 | 18.0, 50.0 |
| Height (cm) |  |  |  |  |
| Mean (SD) | 164.4 (10.0) | 166.4 (10.7) | 165.4 (9.7) | 166.0 (10.4) |
| Median | 163.5 | 165.5 | 165.8 | 167.0 |
| Min, Max | 145.3, 187.0 | 144.5, 192.0 | 144.0, 183.0 | 135.7, 188.0 |
| Weight (kg) |  |  |  |  |
| Mean (SD) | 58.2 (9.9) | 58.7 (11.0) | 59.3 (11.2) | 57.7 (9.6) |
| Median | 58.0 | 57.2 | 58.6 | 57.1 |
| Min, Max | 35.3, 87.6 | 37.9, 99.0 | 40.6, 111.1 | 35.8, 76.9 |
| BMI (kg/m^2^) |  |  |  |  |
| Mean (SD) | 21.5 (3.3) | 21.2 (3.7) | 21.7 (3.8) | 20.9 (3.0) |
| Median | 20.6 | 20.4 | 20.8 | 20.2 |
| Min, Max | 16.0, 34.6 | 16.1, 37.6 | 16.8, 41.8 | 16.6, 31.5 |
| Serostatus to SARS-CoV2 Antibodies, , n (%) |  |  |  |  |
| Seropositive | 72 (82.8%) | 72 (84.7%) | 68 (86.1%) | 72 (86.7%) |
| Seronegative | 15 (17.2%) | 13 (15.3%) | 11 (13.9%) | 11 (13.3%) |
| Abbreviations: A1: BBIBP-CorV, Ad26.COV2.S; A2: BBIBP-CorV, BBIBP-CorV; B1: Ad26.COV2.S, BBIBP-CorV; B2: Placebo, Ad26.COV2.S; N: number of total participants; n: number of participants in the category; %: percentages (100*n/N); SD: Standard Deviation; Min.: Minimum value; Max.: Maximum value; BMI: Body mass index. | | | | |

**Supplementary Table 3.** Geometric Mean Titers, Geometric Mean-Fold Rises and Geometric Mean Ratio of the neutralizing antibodies against SARS-Cov-2 Omicron variant BA.1, (full analysis set).

| **Parameter** | **Time point** | **Arm A1 (BBIBP-CorV, Ad26.COV2.S) N=89** | | **Arm A2 (BBIBP-CorV, BBIBP-CorV) N=90** | | **Arm B1 (Ad26.COV2.S, BBIBP-CorV) N=83** | | **Arm B2 (Placebo, Ad26.COV2.S) n=86** | |
| --- | --- | --- | --- | --- | --- | --- | --- | --- | --- |
|  |  | **Value (95% CI)** | **SD** | **Value (95% CI)** | **SD** | **Value (95% CI)** | **SD** | **Value (95% CI)** | SD |
| **GMT** | Baseline | 180.9 (133.6, 245.0) | 4.2 | 144.3 (100.5, 207.2) | 5.6 | 164.8 (114.6, 236.8) | 5.27 | 179.9 (124.4, 260.1) | 5.6 |
|  | 4 Weeks | 250.6 (187.0, 335.9) | 4 | 195.7 (149.2, 256.5) | 3.6 | 589.9 (437.9, 794.7) | 3.95 | 155.7 (107.3, 225.8) | 5.7 |
|  | 8 weeks | 795.4 (632.6, 1000.0) | 3 | 194.6 (146.6, 258.2) | 3.9 | 613.0 (457.6, 821.2) | 3.82 | 721.4 (533.8, 974.9) | 4 |
| **GMFR** | 4 Weeks | 1.4 (1.2, 1.7) | 2.4 | 1.4 (1.08, 1.7) | 2.9 | 3.52 (2.67, 4.65) | 3.58 | 0.9 (0.75, 1.00) ^†^ | 2 |
|  | 8 weeks | 4.4 (3.4, 5.6) | 3.2 | 1.4 (1.04, 1.7) | 3.4 | 3.61 (2.78, 4.70) | 3.31 | 4.6 (3.5, 6.0) | 3.4 |
| **GMR** | Time point | Crude ratio (A1/A2) | | Adjusted ratio (A1/A2) | | Crude ratio (B1/B2) | | Adjusted ratio (B1/B2) | |
|  | 4 Weeks | 1.3 (0.9, 1.9) | | 1.3 (0.9, 1.9) | | 3.8 (2.4, 6.1) ‡ | | 3.8 (2.5, 5.9) ‡ | |
|  | 8 weeks | 4.1 (2.9, 5.9) | | 4.3 (3.0, 6.1) | | 0.85 (0.6, 1.3) | | 0.84 (0.6, 1.3) | |
| Abbreviations: BBIBP-CorV: Sinopharm vaccine, Ad26.COV2.S: Johnson & Johnson vaccine; N: number of total participants; SD: Geometric Standard Deviation for log-transformed (base 2) titer for the GMT and Geometric Standard Deviation for log-transformed (base 2) fold-rised titer from baseline for the GMFR; GMT: Geometric Mean Titers, GMFR: Geometric Mean Fold Rise, GMR: Geometric Mean ratio (GMTHeteto/GMTHomo). | | | | | | | | | |
| * GMFR Value is calculated from Week 4, not Week 0 for B2(Placebo, Ad26.COV2.S) | | | | | | | | | |
| †The adjusted GMR and 95% confidence interval are derived using the generalized linear regression model by adjusting for age strata (less than 50 years, 50+years), baseline SARS-CoV-2 serostatus, cohort (general/immunology subset) as randomization design variable. ‡ GMR comparison between Ad26.COV2.S vs Placebo | | | | | | | | | |

**Supplementary Table 4.** Local and Systemic solicited reactions occurred respectively within 7 days and 14 days post vaccination by study arms

|  | A1 | | A2 | | B1 | | B2 | |
| --- | --- | --- | --- | --- | --- | --- | --- | --- |
|  | N=93 | | N=91 | | N=90 | | N=93 | |
|  | m | n (%) | m | n (%) | m | n (%) | m | n (%) |
| **Post first dose** |  |  |  |  |  |  |  |  |
| **Solicited AE** | **103** | **27 (29.0%)** | **100** | **35 (38.5%)** | **184** | **48 (53.3%)** | **101** | **31 (33.3%)** |
| **Local AE** | **21** | **16 (17.2%)** | **26** | **19 (20.9%)** | **51** | **35 (38.9%)** | **22** | **19 (20.4%)** |
| Pain | 14 | 14 (15.1%) | 18 | 17 (18.7%) | 35 | 34 (37.8%) | 19 | 18 (19.4%) |
| Redness | 1 | 1 (1.1%) | 2 | 2 (2.2%) | 3 | 3 (3.3%) | 0 | 0 (0.0%) |
| Swelling | 2 | 2 (2.2%) | 4 | 4 (4.4%) | 7 | 7 (7.8%) | 0 | 0 (0.0%) |
| Induration | 1 | 1 (1.1%) | 2 | 2 (2.2%) | 4 | 4 (4.4%) | 0 | 0 (0.0%) |
| Pruritus (Itchiness) | 3 | 3 (3.2%) | 0 | 0 (0.0%) | 2 | 2 (2.2%) | 3 | 3 (3.2%) |
| **Systemic AE** | **82** | **21 (22.6%)** | **74** | **28 (30.8%)** | **133** | **38 (42.2%)** | **79** | **25 (26.9%)** |
| Fever | 6 | 5 (5.4%) | 5 | 3 (3.3%) | 6 | 5 (5.6%) | 2 | 1 (1.1%) |
| Headache | 15 | 12 (12.9%) | 25 | 20 (22.0%) | 37 | 23 (25.6%) | 21 | 16 (17.2%) |
| Fatigue | 10 | 7 (7.5%) | 16 | 13 (14.3%) | 26 | 21 (23.3%) | 13 | 11 (11.8%) |
| Myalgia | 11 | 7 (7.5%) | 6 | 5 (5.5%) | 24 | 19 (21.1%) | 12 | 9 (9.7%) |
| Nausea | 6 | 5 (5.4%) | 1 | 1 (1.1%) | 4 | 4 (4.4%) | 8 | 6 (6.5%) |
| Cough | 14 | 11 (11.8%) | 6 | 5 (5.5%) | 6 | 4 (4.4%) | 8 | 8 (8.6%) |
| Dyspnea | 2 | 2 (2.2%) | 3 | 2 (2.2%) | 0 | 0 (0.0%) | 1 | 1 (1.1%) |
| Diarrhea | 6 | 4 (4.3%) | 3 | 3 (3.3%) | 4 | 4 (4.4%) | 5 | 5 (5.4%) |
| Arthralgia | 1 | 1 (1.1%) | 5 | 4 (4.4%) | 15 | 10 (11.1%) | 4 | 3 (3.2%) |
| Dysphagia | 6 | 4 (4.3%) | 1 | 1 (1.1%) | 3 | 3 (3.3%) | 5 | 3 (3.2%) |
| Vomiting | 2 | 2 (2.2%) | 1 | 1 (1.1%) | 3 | 3 (3.3%) | 0 | 0 (0.0%) |
| Constipation | 0 | 0 (0.0%) | 0 | 0 (0.0%) | 1 | 1 (1.1%) | 0 | 0 (0.0%) |
| Pruritus (No skin lesion) | 0 | 0 (0.0%) | 1 | 1 (1.1%) | 1 | 1 (1.1%) | 0 | 0 (0.0%) |
| Anorexia | 3 | 2 (2.2%) | 0 | 0 (0.0%) | 3 | 3 (3.3%) | 0 | 0 (0.0%) |
| Hypersensitivity reaction | 0 | 0 (0.0%) | 1 | 1 (1.1%) | 0 | 0 (0.0%) | 0 | 0 (0.0%) |
| **Post second dose** |  |  |  |  |  |  |  |  |
| **Solicited AE** | **97** | **34 (38.2%)** | **73** | **24 (26.7%)** | **48** | **17 (20.2%)** | **124** | **35 (40.7%)** |
| **Local AE** | **34** | **30 (33.7%)** | **18** | **16 (17.8%)** | **16** | **11 (13.1%)** | **34** | **23 (26.7%)** |
| Pain | 26 | 26 (29.2%) | 14 | 14 (15.6%) | 11 | 11 (13.1%) | 24 | 23 (26.7%) |
| Redness | 2 | 2 (2.2%) | 1 | 1 (1.1%) | 1 | 1 (1.2%) | 2 | 2 (2.3%) |
| Swelling | 2 | 2 (2.2%) | 2 | 2 (2.2%) | 2 | 2 (2.4%) | 3 | 3 (3.5%) |
| Induration | 1 | 1 (1.1%) | 0 | 0 (0.0%) | 0 | 0 (0.0%) | 3 | 3 (3.5%) |
| Pruritus (Itchiness) | 3 | 3 (3.4%) | 1 | 1 (1.1%) | 2 | 2 (2.4%) | 2 | 2 (2.3%) |
| **Systemic AE** | **63** | **23 (25.8%)** | **55** | **18 (20.0%)** | **32** | **13 (15.5%)** | **90** | **26 (30.2%)** |
| Fever | 7 | 7 (7.9%) | 5 | 3 (3.3%) | 2 | 2 (2.4%) | 11 | 9 (10.5%) |
| Headache | 15 | 15 (16.9%) | 16 | 14 (15.6%) | 4 | 4 (4.8%) | 24 | 20 (23.3%) |
| Fatigue | 13 | 12 (13.5%) | 7 | 6 (6.7%) | 9 | 7 (8.3%) | 12 | 12 (14.0%) |
| Myalgia | 10 | 8 (9.0%) | 6 | 5 (5.6%) | 5 | 4 (4.8%) | 15 | 13 (15.1%) |
| Nausea | 2 | 2 (2.2%) | 5 | 5 (5.6%) | 2 | 2 (2.4%) | 7 | 5 (5.8%) |
| Cough | 7 | 6 (6.7%) | 6 | 6 (6.7%) | 7 | 6 (7.1%) | 5 | 4 (4.7%) |
| Dyspnea | 0 | 0 (0.0%) | 1 | 1 (1.1%) | 0 | 0 (0.0%) | 1 | 1 (1.2%) |
| Diarrhea | 1 | 1 (1.1%) | 4 | 4 (4.4%) | 0 | 0 (0.0%) | 0 | 0 (0.0%) |
| Arthralgia | 3 | 3 (3.4%) | 1 | 1 (1.1%) | 2 | 2 (2.4%) | 6 | 4 (4.7%) |
| Dysphagia | 4 | 2 (2.2%) | 2 | 2 (2.2%) | 0 | 0 (0.0%) | 4 | 3 (3.5%) |
| Vomiting | 0 | 0 (0.0%) | 2 | 2 (2.2%) | 1 | 1 (1.2%) | 1 | 1 (1.2%) |
| Constipation | 0 | 0 (0.0%) | 0 | 0 (0.0%) | 0 | 0 (0.0%) | 2 | 1 (1.2%) |
| Pruritus (No skin lesion) | 0 | 0 (0.0%) | 0 | 0 (0.0%) | 0 | 0 (0.0%) | 0 | 0 (0.0%) |
| Anorexia | 0 | 0 (0.0%) | 0 | 0 (0.0%) | 0 | 0 (0.0%) | 2 | 1 (1.2%) |
| Hypersensitivity reaction | 1 | 1 (1.1%) | 0 | 0 (0.0%) | 0 | 0 (0.0%) | 0 | 0 (0.0%) |
| **Post any dose** |  |  |  |  |  |  |  |  |
| **Solicited AE** | **200** | **43 (46.2%)** | **173** | **48 (52.7%)** | **232** | **50 (55.6%)** | **225** | **48 (51.6%)** |
| **Local AE** | **55** | **38 (40.9%)** | **44** | **28 (30.8%)** | **67** | **36 (40.0%)** | **56** | **29 (31.2%)** |
| Pain | 40 | 34 (36.6%) | 32 | 25 (27.5%) | 46 | 36 (40.0%) | 43 | 29 (31.2%) |
| Redness | 3 | 3 (3.2%) | 3 | 3 (3.3%) | 4 | 4 (4.4%) | 2 | 2 (2.2%) |
| Swelling | 4 | 4 (4.3%) | 6 | 4 (4.4%) | 9 | 7 (7.8%) | 3 | 3 (3.2%) |
| Induration | 2 | 2 (2.2%) | 2 | 2 (2.2%) | 4 | 4 (4.4%) | 3 | 3 (3.2%) |
| Pruritus (Itchiness) | 6 | 6 (6.5%) | 1 | 1 (1.1%) | 4 | 4 (4.4%) | 5 | 4 (4.3%) |
| **Systemic AE** | **145** | **36 (38.7%)** | **129** | **39 (42.9%)** | **165** | **41 (45.6%)** | **169** | **40 (43.0%)** |
| Fever | 13 | 11 (11.8%) | 10 | 5 (5.5%) | 8 | 7 (7.8%) | 13 | 9 (9.7%) |
| Headache | 30 | 25 (26.9%) | 41 | 31 (34.1%) | 41 | 24 (26.7%) | 45 | 31 (33.3%) |
| Fatigue | 23 | 18 (19.4%) | 23 | 18 (19.8%) | 35 | 23 (25.6%) | 25 | 19 (20.4%) |
| Myalgia | 21 | 14 (15.1%) | 12 | 10 (11.0%) | 29 | 21 (23.3%) | 27 | 19 (20.4%) |
| Nausea | 8 | 7 (7.5%) | 6 | 6 (6.6%) | 6 | 6 (6.7%) | 15 | 9 (9.7%) |
| Cough | 21 | 15 (16.1%) | 12 | 10 (11.0%) | 13 | 10 (11.1%) | 13 | 10 (10.8%) |
| Dyspnea | 2 | 2 (2.2%) | 4 | 3 (3.3%) | 0 | 0 (0.0%) | 2 | 2 (2.2%) |
| Diarrhea | 7 | 4 (4.3%) | 7 | 5 (5.5%) | 4 | 4 (4.4%) | 5 | 5 (5.4%) |
| Arthralgia | 4 | 3 (3.2%) | 6 | 5 (5.5%) | 17 | 11 (12.2%) | 10 | 6 (6.5%) |
| Dysphagia | 10 | 6 (6.5%) | 3 | 3 (3.3%) | 3 | 3 (3.3%) | 9 | 4 (4.3%) |
| Vomiting | 2 | 2 (2.2%) | 3 | 3 (3.3%) | 4 | 4 (4.4%) | 1 | 1 (1.1%) |
| Constipation | 0 | 0 (0.0%) | 0 | 0 (0.0%) | 1 | 1 (1.1%) | 2 | 1 (1.1%) |
| Pruritus (No skin lesion) | 0 | 0 (0.0%) | 1 | 1 (1.1%) | 1 | 1 (1.1%) | 0 | 0 (0.0%) |
| Anorexia | 3 | 2 (2.2%) | 0 | 0 (0.0%) | 3 | 3 (3.3%) | 2 | 1 (1.1%) |
| Hypersensitivity reaction | 1 | 1 (1.1%) | 1 | 1 (1.1%) | 0 | 0 (0.0%) | 0 | 0 (0.0%) |

**Supplementary Table 5.** Local and Systemic solicited reactions occurred respectively within 7 days and 14 days post vaccination with severity by study arms

|  | A1 | A2 | B1 | B2 |
| --- | --- | --- | --- | --- |
|  | N=93 | N=91 | N=90 | N=93 |
| **Post first dose (number of events)** |  |  |  |  |
| **Solicited AE** | 103 | 100 | 184 | 101 |
| **-Mild** | 80 (77.7%) | 79 (79.0%) | 145 (78.8%) | 89 (88.1%) |
| **-Moderate** | 21 (20.4%) | 19 (19.0%) | 37 (20.1%) | 12 (11.9%) |
| **-Severe** | 2 (1.9%) | 2 (2.0%) | 2 (1.1%) | 0 (0.0%) |
| **Local AE** | 21 | 26 | 51 | 22 |
| **-Mild** | 19 (90.5%) | 24 (92.3%) | 38 (74.5%) | 19 (86.4%) |
| **-Moderate** | 2 (9.5%) | 2 (7.7%) | 12 (23.5%) | 3 (13.6%) |
| **-Severe** | 0 (0.0%) | 0 (0.0%) | 1 (2.0%) | 0 (0.0%) |
| Pain | 14 | 18 | 35 | 19 |
| -Mild | 12 (85.7%) | 17 (94.4%) | 28 (80.0%) | 16 (84.2%) |
| -Moderate | 2 (14.3%) | 1 (5.6%) | 6 (17.1%) | 3 (15.8%) |
| -Severe | 0 (0.0%) | 0 (0.0%) | 1 (2.9%) | 0 (0.0%) |
| Redness | 1 | 2 | 3 | 0 |
| -Mild | 1 (100.0%) | 2 (100.0%) | 2 (66.7%) | 0 (0.0%) |
| -Moderate | 0 (0.0%) | 0 (0.0%) | 1 (33.3%) | 0 (0.0%) |
| -Severe | 0 (0.0%) | 0 (0.0%) | 0 (0.0%) | 0 (0.0%) |
| Swelling | 2 | 4 | 7 | 0 |
| -Mild | 2 (100.0%) | 3 (75.0%) | 4 (57.1%) | 0 (0.0%) |
| -Moderate | 0 (0.0%) | 1 (25.0%) | 3 (42.9%) | 0 (0.0%) |
| -Severe | 0 (0.0%) | 0 (0.0%) | 0 (0.0%) | 0 (0.0%) |
| Induration | 1 | 2 | 4 | 0 |
| -Mild | 1 (100.0%) | 2 (100.0%) | 2 (50.0%) | 0 (0.0%) |
| -Moderate | 0 (0.0%) | 0 (0.0%) | 2 (50.0%) | 0 (0.0%) |
| -Severe | 0 (0.0%) | 0 (0.0%) | 0 (0.0%) | 0 (0.0%) |
| Pruritus (Itchiness) | 3 | 0 | 2 | 3 |
| -Mild | 3 (100.0%) | 0 (0.0%) | 2 (100.0%) | 3 (100.0%) |
| -Moderate | 0 (0.0%) | 0 (0.0%) | 0 (0.0%) | 0 (0.0%) |
| -Severe | 0 (0.0%) | 0 (0.0%) | 0 (0.0%) | 0 (0.0%) |
| **Systemic AE** | 82 | 74 | 133 | 79 |
| **-Mild** | 61 (74.4%) | 55 (74.3%) | 107 (80.5%) | 70 (88.6%) |
| **-Moderate** | 19 (23.2%) | 17 (23.0%) | 25 (18.8%) | 9 (11.4%) |
| **-Severe** | 2 (2.4%) | 2 (2.7%) | 1 (0.8%) | 0 (0.0%) |
| Fever | 6 | 5 | 6 | 2 |
| -Mild | 4 (66.7%) | 2 (40.0%) | 4 (66.7%) | 1 (50.0%) |
| -Moderate | 2 (33.3%) | 2 (40.0%) | 1 (16.7%) | 1 (50.0%) |
| -Severe | 0 (0.0%) | 1 (20.0%) | 1 (16.7%) | 0 (0.0%) |
| Headache | 15 | 25 | 37 | 21 |
| -Mild | 10 (66.7%) | 19 (76.0%) | 28 (75.7%) | 19 (90.5%) |
| -Moderate | 4 (26.7%) | 6 (24.0%) | 9 (24.3%) | 2 (9.5%) |
| -Severe | 1 (6.7%) | 0 (0.0%) | 0 (0.0%) | 0 (0.0%) |
| Fatigue | 10 | 16 | 26 | 13 |
| -Mild | 7 (70.0%) | 10 (62.5%) | 22 (84.6%) | 12 (92.3%) |
| -Moderate | 3 (30.0%) | 5 (31.3%) | 4 (15.4%) | 1 (7.7%) |
| -Severe | 0 (0.0%) | 1 (6.3%) | 0 (0.0%) | 0 (0.0%) |
| Myalgia | 11 | 6 | 24 | 12 |
| -Mild | 10 (90.9%) | 5 (83.3%) | 17 (70.8%) | 11 (91.7%) |
| -Moderate | 1 (9.1%) | 1 (16.7%) | 7 (29.2%) | 1 (8.3%) |
| -Severe | 0 (0.0%) | 0 (0.0%) | 0 (0.0%) | 0 (0.0%) |
| Nausea | 6 | 1 | 4 | 8 |
| -Mild | 5 (83.3%) | 1 (100.0%) | 4 (100.0%) | 8 (100.0%) |
| -Moderate | 0 (0.0%) | 0 (0.0%) | 0 (0.0%) | 0 (0.0%) |
| -Severe | 1 (16.7%) | 0 (0.0%) | 0 (0.0%) | 0 (0.0%) |
| Cough | 14 | 6 | 6 | 8 |
| -Mild | 9 (64.3%) | 6 (100.0%) | 6 (100.0%) | 6 (75.0%) |
| -Moderate | 5 (35.7%) | 0 (0.0%) | 0 (0.0%) | 2 (25.0%) |
| -Severe | 0 (0.0%) | 0 (0.0%) | 0 (0.0%) | 0 (0.0%) |
| Dyspnea | 2 | 3 | 0 | 1 |
| -Mild | 0 (0.0%) | 2 (66.7%) | 0 (0.0%) | 1 (100.0%) |
| -Moderate | 2 (100.0%) | 1 (33.3%) | 0 (0.0%) | 0 (0.0%) |
| -Severe | 0 (0.0%) | 0 (0.0%) | 0 (0.0%) | 0 (0.0%) |
| Diarrhea | 6 | 3 | 4 | 5 |
| -Mild | 6 (100.0%) | 2 (66.7%) | 4 (100.0%) | 4 (80.0%) |
| -Moderate | 0 (0.0%) | 1 (33.3%) | 0 (0.0%) | 1 (20.0%) |
| -Severe | 0 (0.0%) | 0 (0.0%) | 0 (0.0%) | 0 (0.0%) |
| Arthralgia | 1 | 5 | 15 | 4 |
| -Mild | 1 (100.0%) | 4 (80.0%) | 12 (80.0%) | 4 (100.0%) |
| -Moderate | 0 (0.0%) | 1 (20.0%) | 3 (20.0%) | 0 (0.0%) |
| -Severe | 0 (0.0%) | 0 (0.0%) | 0 (0.0%) | 0 (0.0%) |
| Dysphagia | 6 | 1 | 3 | 5 |
| -Mild | 5 (83.3%) | 1 (100.0%) | 3 (100.0%) | 4 (80.0%) |
| -Moderate | 1 (16.7%) | 0 (0.0%) | 0 (0.0%) | 1 (20.0%) |
| -Severe | 0 (0.0%) | 0 (0.0%) | 0 (0.0%) | 0 (0.0%) |
| Vomiting | 2 | 1 | 3 | 0 |
| -Mild | 2 (100.0%) | 1 (100.0%) | 3 (100.0%) | 0 (0.0%) |
| -Moderate | 0 (0.0%) | 0 (0.0%) | 0 (0.0%) | 0 (0.0%) |
| -Severe | 0 (0.0%) | 0 (0.0%) | 0 (0.0%) | 0 (0.0%) |
| Constipation | 0 | 0 | 1 | 0 |
| -Mild | 0 (0.0%) | 0 (0.0%) | 1 (100.0%) | 0 (0.0%) |
| -Moderate | 0 (0.0%) | 0 (0.0%) | 0 (0.0%) | 0 (0.0%) |
| -Severe | 0 (0.0%) | 0 (0.0%) | 0 (0.0%) | 0 (0.0%) |
| Pruritus (No skin lesion) | 0 | 1 | 1 | 0. |
| -Mild | 0 (0.0%) | 1 (100.0%) | 1 (100.0%) | 0 (0.0%) |
| -Moderate | 0 (0.0%) | 0 (0.0%) | 0 (0.0%) | 0 (0.0%) |
| -Severe | 0 (0.0%) | 0 (0.0%) | 0 (0.0%) | 0 (0.0%) |
| Anorexia | 3 | 0 | 3 | 0 |
| -Mild | 2 (66.7%) | 0 (0.0%) | 2 (66.7%) | 0 (0.0%) |
| -Moderate | 1 (33.3%) | 0 (0.0%) | 1 (33.3%) | 0 (0.0%) |
| -Severe | 0 (0.0%) | 0 (0.0%) | 0 (0.0%) | 0 (0.0%) |
| Hypersensitivity reaction | 0 | 1 | 0 | 0 |
| -Mild | 0 (0.0%) | 1 (100.0%) | 0 (0.0%) | 0 (0.0%) |
| -Moderate | 0 (0.0%) | 0 (0.0%) | 0 (0.0%) | 0 (0.0%) |
| -Severe | 0 (0.0%) | 0 (0.0%) | 0 (0.0%) | 0 (0.0%) |

**[Second dose]**

|  | A1 | A2 | B1 | B2 |
| --- | --- | --- | --- | --- |
|  | N=89 | N=90 | N=84 | N=86 |
| **Post second dose (number of events)** |  |  |  |  |
| **Solicited AE** | 97 | 73 | 48 | 124 |
| **-Mild** | 67 (69.1%) | 51 (69.9%) | 40 (83.3%) | 94 (75.8%) |
| **-Moderate** | 30 (30.9%) | 19 (26.0%) | 8 (16.7%) | 30 (24.2%) |
| **-Severe** | 0 (0.0%) | 3 (4.1%) | 0 (0.0%) | 0 (0.0%) |
| **Local AE** | 34 | 18 | 16 | 34 |
| **-Mild** | 25 (73.5%) | 14 (77.8%) | 10 (62.5%) | 24 (70.6%) |
| **-Moderate** | 9 (26.5%) | 4 (22.2%) | 6 (37.5%) | 10 (29.4%) |
| **-Severe** | 0 (0.0%) | 0 (0.0%) | 0 (0.0%) | 0 (0.0%) |
| Pain | 26 | 14 | 11 | 24 |
| -Mild | 17 (65.4%) | 10 (71.4%) | 8 (72.7%) | 14 (58.3%) |
| -Moderate | 9 (34.6%) | 4 (28.6%) | 3 (27.3%) | 10 (41.7%) |
| -Severe | 0 (0.0%) | 0 (0.0%) | 0 (0.0%) | 0 (0.0%) |
| Redness | 2 | 1 | 1 | 2 |
| -Mild | 2 (100.0%) | 1 (100.0%) | 1 (100.0%) | 2 (100.0%) |
| -Moderate | 0 (0.0%) | 0 (0.0%) | 0 (0.0%) | 0 (0.0%) |
| -Severe | 0 (0.0%) | 0 (0.0%) | 0 (0.0%) | 0 (0.0%) |
| Swelling | 2 | 2 | 2 | 3 |
| -Mild | 2 (100.0%) | 2 (100.0%) | 1 (50.0%) | 3 (100.0%) |
| -Moderate | 0 (0.0%) | 0 (0.0%) | 1 (50.0%) | 0 (0.0%) |
| -Severe | 0 (0.0%) | 0 (0.0%) | 0 (0.0%) | 0 (0.0%) |
| Induration | 1 | 0 | 0 | 3 |
| -Mild | 1 (100.0%) | 0 (0.0%) | 0 (0.0%) | 3 (100.0%) |
| -Moderate | 0 (0.0%) | 0 (0.0%) | 0 (0.0%) | 0 (0.0%) |
| -Severe | 0 (0.0%) | 0 (0.0%) | 0 (0.0%) | 0 (0.0%) |
| Pruritus (Itchiness) | 3 | 1 | 2 | 2 |
| -Mild | 3 (100.0%) | 1 (100.0%) | 0 (0.0%) | 2 (100.0%) |
| -Moderate | 0 (0.0%) | 0 (0.0%) | 2 (100.0%) | 0 (0.0%) |
| -Severe | 0 (0.0%) | 0 (0.0%) | 0 (0.0%) | 0 (0.0%) |
| **Systemic AE** | 63 | 55 | 32 | 90 |
| **-Mild** | 42 (66.7%) | 37 (67.3%) | 30 (93.8%) | 70 (77.8%) |
| **-Moderate** | 21 (33.3%) | 15 (27.3%) | 2 (6.3%) | 20 (22.2%) |
| **-Severe** | 0 (0.0%) | 3 (5.5%) | 0 (0.0%) | 0 (0.0%) |
| Fever | 7 | 5 | 2 | 11 |
| -Mild | 6 (85.7%) | 1 (20.0%) | 2 (100.0%) | 9 (81.8%) |
| -Moderate | 1 (14.3%) | 1 (20.0%) | 0 (0.0%) | 2 (18.2%) |
| -Severe | 0 (0.0%) | 3 (60.0%) | 0 (0.0%) | 0 (0.0%) |
| Headache | 15 | 16 | 4 | 24 |
| -Mild | 9 (60.0%) | 11 (68.8%) | 3 (75.0%) | 16 (66.7%) |
| -Moderate | 6 (40.0%) | 5 (31.3%) | 1 (25.0%) | 8 (33.3%) |
| -Severe | 0 (0.0%) | 0 (0.0%) | 0 (0.0%) | 0 (0.0%) |
| Fatigue | 13 | 7 | 9 | 12 |
| -Mild | 6 (46.2%) | 5 (71.4%) | 8 (88.9%) | 8 (66.7%) |
| -Moderate | 7 (53.8%) | 2 (28.6%) | 1 (11.1%) | 4 (33.3%) |
| -Severe | 0 (0.0%) | 0 (0.0%) | 0 (0.0%) | 0 (0.0%) |
| Myalgia | 10 | 6 | 5 | 15 |
| -Mild | 6 (60.0%) | 4 (66.7%) | 5 (100.0%) | 10 (66.7%) |
| -Moderate | 4 (40.0%) | 2 (33.3%) | 0 (0.0%) | 5 (33.3%) |
| -Severe | 0 (0.0%) | 0 (0.0%) | 0 (0.0%) | 0 (0.0%) |
| Nausea | 2 | 5 | 2 | 7 |
| -Mild | 2 (100.0%) | 3 (60.0%) | 2 (100.0%) | 7 (100.0%) |
| -Moderate | 0 (0.0%) | 2 (40.0%) | 0 (0.0%) | 0 (0.0%) |
| -Severe | 0 (0.0%) | 0 (0.0%) | 0 (0.0%) | 0 (0.0%) |
| Cough | 7 | 6 | 7 | 5 |
| -Mild | 7 (100.0%) | 6 (100.0%) | 7 (100.0%) | 5 (100.0%) |
| -Moderate | 0 (0.0%) | 0 (0.0%) | 0 (0.0%) | 0 (0.0%) |
| -Severe | 0 (0.0%) | 0 (0.0%) | 0 (0.0%) | 0 (0.0%) |
| Dyspnea | 0 | 1 | 0 | 1 |
| -Mild | 0 (0.0%) | 0 (0.0%) | 0 (0.0%) | 1 (100.0%) |
| -Moderate | 0 (0.0%) | 1 (100.0%) | 0 (0.0%) | 0 (0.0%) |
| -Severe | 0 (0.0%) | 0 (0.0%) | 0 (0.0%) | 0 (0.0%) |
| Diarrhea | 1 | 4 | 0 | 0 |
| -Mild | 0 (0.0%) | 4 (100.0%) | 0 (0.0%) | 0 (0.0%) |
| -Moderate | 1 (100.0%) | 0 (0.0%) | 0 (0.0%) | 0 (0.0%) |
| -Severe | 0 (0.0%) | 0 (0.0%) | 0 (0.0%) | 0 (0.0%) |
| Arthralgia | 3 | 1 | 2 | 6 |
| -Mild | 1 (33.3%) | 1 (100.0%) | 2 (100.0%) | 5 (83.3%) |
| -Moderate | 2 (66.7%) | 0 (0.0%) | 0 (0.0%) | 1 (16.7%) |
| -Severe | 0 (0.0%) | 0 (0.0%) | 0 (0.0%) | 0 (0.0%) |
| Dysphagia | 4 | 2 | 0 | 4 |
| -Mild | 4 (100.0%) | 2 (100.0%) | 0 (0.0%) | 4 (100.0%) |
| -Moderate | 0 (0.0%) | 0 (0.0%) | 0 (0.0%) | 0 (0.0%) |
| -Severe | 0 (0.0%) | 0 (0.0%) | 0 (0.0%) | 0 (0.0%) |
| Vomiting | 0 | 2 | 1 | 1 |
| -Mild | 0 (0.0%) | 0 (0.0%) | 1 (100.0%) | 1 (100.0%) |
| -Moderate | 0 (0.0%) | 2 (100.0%) | 0 (0.0%) | 0 (0.0%) |
| -Severe | 0 (0.0%) | 0 (0.0%) | 0 (0.0%) | 0 (0.0%) |
| Constipation | 0 | 0 | 0 | 2 |
| -Mild | 0 (0.0%) | 0 (0.0%) | 0 (0.0%) | 2 (100.0%) |
| -Moderate | 0 (0.0%) | 0 (0.0%) | 0 (0.0%) | 0 (0.0%) |
| -Severe | 0 (0.0%) | 0 (0.0%) | 0 (0.0%) | 0 (0.0%) |
| Pruritus (No skin lesion) | 0 | 0 | 0 | 0 |
| -Mild | 0 (0.0%) | 0 (0.0%) | 0 (0.0%) | 0 (0.0%) |
| -Moderate | 0 (0.0%) | 0 (0.0%) | 0 (0.0%) | 0 (0.0%) |
| -Severe | 0 (0.0%) | 0 (0.0%) | 0 (0.0%) | 0 (0.0%) |
| Anorexia | 0 | 0 | 0 | 2 |
| -Mild | 0 (0.0%) | 0 (0.0%) | 0 (0.0%) | 2 (100.0%) |
| -Moderate | 0 (0.0%) | 0 (0.0%) | 0 (0.0%) | 0 (0.0%) |
| -Severe | 0 (0.0%) | 0 (0.0%) | 0 (0.0%) | 0 (0.0%) |
| Hypersensitivity reaction | 1 | 0 | 0 | 0 |
| -Mild | 1 (100.0%) | 0 (0.0%) | 0 (0.0%) | 0 (0.0%) |
| -Moderate | 0 (0.0%) | 0 (0.0%) | 0 (0.0%) | 0 (0.0%) |
| -Severe | 0 (0.0%) | 0 (0.0%) | 0 (0.0%) | 0 (0.0%) |

**[Any dose]**

|  | A1 | A2 | B1 | B2 |
| --- | --- | --- | --- | --- |
|  | N=93 | N=91 | N=90 | N=93 |
| **Post any dose (number of events)** |  |  |  |  |
| **Solicited AE** | 200 | 173 | 232 | 225 |
| **-Mild** | 147 (73.5%) | 130 (75.1%) | 185 (79.7%) | 183 (81.3%) |
| **-Moderate** | 51 (25.5%) | 38 (22.0%) | 45 (19.4%) | 42 (18.7%) |
| **-Severe** | 2 (1.0%) | 5 (2.9%) | 2 (0.9%) | 0 (0.0%) |
| **Local AE** | 55 | 44 | 67 | 56 |
| **-Mild** | 44 (80.0%) | 38 (86.4%) | 48 (71.6%) | 43 (76.8%) |
| **-Moderate** | 11 (20.0%) | 6 (13.6%) | 18 (26.9%) | 13 (23.2%) |
| **-Severe** | 0 (0.0%) | 0 (0.0%) | 1 (1.5%) | 0 (0.0%) |
| Pain | 40 | 32 | 46 | 43 |
| -Mild | 29 (72.5%) | 27 (84.4%) | 36 (78.3%) | 30 (69.8%) |
| -Moderate | 11 (27.5%) | 5 (15.6%) | 9 (19.6%) | 13 (30.2%) |
| -Severe | 0 (0.0%) | 0 (0.0%) | 1 (2.2%) | 0 (0.0%) |
| Redness | 3 | 3 | 4 | 2 |
| -Mild | 3 (100.0%) | 3 (100.0%) | 3 (75.0%) | 2 (100.0%) |
| -Moderate | 0 (0.0%) | 0 (0.0%) | 1 (25.0%) | 0 (0.0%) |
| -Severe | 0 (0.0%) | 0 (0.0%) | 0 (0.0%) | 0 (0.0%) |
| Swelling | 4 | 6 | 9 | 3 |
| -Mild | 4 (100.0%) | 5 (83.3%) | 5 (55.6%) | 3 (100.0%) |
| -Moderate | 0 (0.0%) | 1 (16.7%) | 4 (44.4%) | 0 (0.0%) |
| -Severe | 0 (0.0%) | 0 (0.0%) | 0 (0.0%) | 0 (0.0%) |
| Induration | 2 | 2 | 4 | 3 |
| -Mild | 2 (100.0%) | 2 (100.0%) | 2 (50.0%) | 3 (100.0%) |
| -Moderate | 0 (0.0%) | 0 (0.0%) | 2 (50.0%) | 0 (0.0%) |
| -Severe | 0 (0.0%) | 0 (0.0%) | 0 (0.0%) | 0 (0.0%) |
| Pruritus (Itchiness) | 6 | 1 | 4 | 5 |
| -Mild | 6 (100.0%) | 1 (100.0%) | 2 (50.0%) | 5 (100.0%) |
| -Moderate | 0 (0.0%) | 0 (0.0%) | 2 (50.0%) | 0 (0.0%) |
| -Severe | 0 (0.0%) | 0 (0.0%) | 0 (0.0%) | 0 (0.0%) |
| **Systemic AE** | 145 | 129 | 165 | 169 |
| **-Mild** | 103 (71.0%) | 92 (71.3%) | 137 (83.0%) | 140 (82.8%) |
| **-Moderate** | 40 (27.6%) | 32 (24.8%) | 27 (16.4%) | 29 (17.2%) |
| **-Severe** | 2 (1.4%) | 5 (3.9%) | 1 (0.6%) | 0 (0.0%) |
| Fever | 13 | 10 | 8 | 13 |
| -Mild | 10 (76.9%) | 3 (30.0%) | 6 (75.0%) | 10 (76.9%) |
| -Moderate | 3 (23.1%) | 3 (30.0%) | 1 (12.5%) | 3 (23.1%) |
| -Severe | 0 (0.0%) | 4 (40.0%) | 1 (12.5%) | 0 (0.0%) |
| Headache | 30 | 41 | 41 | 45 |
| -Mild | 19 (63.3%) | 30 (73.2%) | 31 (75.6%) | 35 (77.8%) |
| -Moderate | 10 (33.3%) | 11 (26.8%) | 10 (24.4%) | 10 (22.2%) |
| -Severe | 1 (3.3%) | 0 (0.0%) | 0 (0.0%) | 0 (0.0%) |
| Fatigue | 23 | 23 | 35 | 25 |
| -Mild | 13 (56.5%) | 15 (65.2%) | 30 (85.7%) | 20 (80.0%) |
| -Moderate | 10 (43.5%) | 7 (30.4%) | 5 (14.3%) | 5 (20.0%) |
| -Severe | 0 (0.0%) | 1 (4.3%) | 0 (0.0%) | 0 (0.0%) |
| Myalgia | 21 | 12 | 29 | 27 |
| -Mild | 16 (76.2%) | 9 (75.0%) | 22 (75.9%) | 21 (77.8%) |
| -Moderate | 5 (23.8%) | 3 (25.0%) | 7 (24.1%) | 6 (22.2%) |
| -Severe | 0 (0.0%) | 0 (0.0%) | 0 (0.0%) | 0 (0.0%) |
| Nausea | 8 | 6 | 6 | 15 |
| -Mild | 7 (87.5%) | 4 (66.7%) | 6 (100.0%) | 15 (100.0%) |
| -Moderate | 0 (0.0%) | 2 (33.3%) | 0 (0.0%) | 0 (0.0%) |
| -Severe | 1 (12.5%) | 0 (0.0%) | 0 (0.0%) | 0 (0.0%) |
| Cough | 21 | 12 | 13 | 13 |
| -Mild | 16 (76.2%) | 12 (100.0%) | 13 (100.0%) | 11 (84.6%) |
| -Moderate | 5 (23.8%) | 0 (0.0%) | 0 (0.0%) | 2 (15.4%) |
| -Severe | 0 (0.0%) | 0 (0.0%) | 0 (0.0%) | 0 (0.0%) |
| Dyspnea | 2 | 4 | 0 | 2 |
| -Mild | 0 (0.0%) | 2 (50.0%) | 0 (0.0%) | 2 (100.0%) |
| -Moderate | 2 (100.0%) | 2 (50.0%) | 0 (0.0%) | 0 (0.0%) |
| -Severe | 0 (0.0%) | 0 (0.0%) | 0 (0.0%) | 0 (0.0%) |
| Diarrhea | 7 | 7 | 4 | 5 |
| -Mild | 6 (85.7%) | 6 (85.7%) | 4 (100.0%) | 4 (80.0%) |
| -Moderate | 1 (14.3%) | 1 (14.3%) | 0 (0.0%) | 1 (20.0%) |
| -Severe | 0 (0.0%) | 0 (0.0%) | 0 (0.0%) | 0 (0.0%) |
| Arthralgia | 4 | 6 | 17 | 10 |
| -Mild | 2 (50.0%) | 5 (83.3%) | 14 (82.4%) | 9 (90.0%) |
| -Moderate | 2 (50.0%) | 1 (16.7%) | 3 (17.6%) | 1 (10.0%) |
| -Severe | 0 (0.0%) | 0 (0.0%) | 0 (0.0%) | 0 (0.0%) |
| Dysphagia | 10 | 3 | 3 | 9 |
| -Mild | 9 (90.0%) | 3 (100.0%) | 3 (100.0%) | 8 (88.9%) |
| -Moderate | 1 (10.0%) | 0 (0.0%) | 0 (0.0%) | 1 (11.1%) |
| -Severe | 0 (0.0%) | 0 (0.0%) | 0 (0.0%) | 0 (0.0%) |
| Vomiting | 2 | 3 | 4 | 1 |
| -Mild | 2 (100.0%) | 1 (33.3%) | 4 (100.0%) | 1 (100.0%) |
| -Moderate | 0 (0.0%) | 2 (66.7%) | 0 (0.0%) | 0 (0.0%) |
| -Severe | 0 (0.0%) | 0 (0.0%) | 0 (0.0%) | 0 (0.0%) |
| Constipation | 0 | 0 | 1 | 2 |
| -Mild | 0 (0.0%) | 0 (0.0%) | 1 (100.0%) | 2 (100.0%) |
| -Moderate | 0 (0.0%) | 0 (0.0%) | 0 (0.0%) | 0 (0.0%) |
| -Severe | 0 (0.0%) | 0 (0.0%) | 0 (0.0%) | 0 (0.0%) |
| Pruritus (No skin lesion) | 0 | 1 | 1 | 0 |
| -Mild | 0 (0.0%) | 1 (100.0%) | 1 (100.0%) | 0 (0.0%) |
| -Moderate | 0 (0.0%) | 0 (0.0%) | 0 (0.0%) | 0 (0.0%) |
| -Severe | 0 (0.0%) | 0 (0.0%) | 0 (0.0%) | 0 (0.0%) |
| Anorexia | 3 | 0 | 3 | 2 |
| -Mild | 2 (66.7%) | 0 (0.0%) | 2 (66.7%) | 2 (100.0%) |
| -Moderate | 1 (33.3%) | 0 (0.0%) | 1 (33.3%) | 0 (0.0%) |
| -Severe | 0 (0.0%) | 0 (0.0%) | 0 (0.0%) | 0 (0.0%) |
| Hypersensitivity reaction | 1 | 1 | 0 | 0 |
| -Mild | 1 (100.0%) | 1 (100.0%) | 0 (0.0%) | 0 (0.0%) |
| -Moderate | 0 (0.0%) | 0 (0.0%) | 0 (0.0%) | 0 (0.0%) |
| -Severe | 0 (0.0%) | 0 (0.0%) | 0 (0.0%) | 0 (0.0%) |

**Supplementary Table 6.** Unsolicited adverse events (SOC/PT) occurred respectively within 28 days post vaccination by study arms

| During 28 days post 1st vaccination | A1 | | A2 | | B1 | | B2 | |
| --- | --- | --- | --- | --- | --- | --- | --- | --- |
|  | N=93 | | N=91 | | N=90 | | N=93 | |
|  | m | n (%) | m | n (%) | m | n (%) | m | n (%) |
| **Unsolicited AE** | **1** | **1 (1.1%)** | **2** | **2 (2.2%)** | **3** | **2 (2.2%)** | **4** | **3 (3.2%)** |
| **Gastrointestinal disorders** | **0** | **0 (0.0%)** | **0** | **0 (0.0%)** | **1** | **1 (1.1%)** | **0** | **0 (0.0%)** |
| Abdominal pain upper | 0 | 0 (0.0%) | 0 | 0 (0.0%) | 1 | 1 (1.1%) | 0 | 0 (0.0%) |
| **Infections and infestations** | **0** | **0 (0.0%)** | **0** | **0 (0.0%)** | **1** | **1 (1.1%)** | **2** | **1 (1.1%)** |
| Nasopharyngitis | 0 | 0 (0.0%) | 0 | 0 (0.0%) | 1 | 1 (1.1%) | 1 | 1 (1.1%) |
| Pharyngitis | 0 | 0 (0.0%) | 0 | 0 (0.0%) | 0 | 0 (0.0%) | 1 | 1 (1.1%) |
| **Injury, poisoning and procedural complications** | **0** | **0 (0.0%)** | **0** | **0 (0.0%)** | **0** | **0 (0.0%)** | **1** | **1 (1.1%)** |
| Joint injury | 0 | 0 (0.0%) | 0 | 0 (0.0%) | 0 | 0 (0.0%) | 1 | 1 (1.1%) |
| **Musculoskeletal and connective tissue disorders** | **0** | **0 (0.0%)** | **0** | **0 (0.0%)** | **0** | **0 (0.0%)** | **1** | **1 (1.1%)** |
| Arthralgia | 0 | 0 (0.0%) | 0 | 0 (0.0%) | 0 | 0 (0.0%) | 1 | 1 (1.1%) |
| **Nervous system disorders** | **0** | **0 (0.0%)** | **1** | **1 (1.1%)** | **0** | **0 (0.0%)** | **0** | **0 (0.0%)** |
| Headache | 0 | 0 (0.0%) | 1 | 1 (1.1%) | 0 | 0 (0.0%) | 0 | 0 (0.0%) |
| **Respiratory, thoracic and mediastinal disorders** | **1** | **1 (1.1%)** | **1** | **1 (1.1%)** | **1** | **1 (1.1%)** | **0** | **0 (0.0%)** |
| Cough | 1 | 1 (1.1%) | 1 | 1 (1.1%) | 1 | 1 (1.1%) | 0 | 0 (0.0%) |
|  |  |  |  |  |  |  |  |  |
| During 28 days post 2nd vaccination | A1 | | A2 | | B1 | | B2 | |
|  | N=89 | | N=90 | | N=84 | | N=86 | |
|  | m | n (%) | m | n (%) | m | n (%) | m | n (%) |
| **Unsolicited AE** | **6** | **4 (4.5%)** | **9** | **5 (5.6%)** | **1** | **1 (1.2%)** | **0** | **0 (0.0%)** |
| **Blood and lymphatic system disorders** | **0** | **0 (0.0%)** | **1** | **1 (1.1%)** | **0** | **0 (0.0%)** | **0** | **0 (0.0%)** |
| Anaemia | 0 | 0 (0.0%) | 1 | 1 (1.1%) | 0 | 0 (0.0%) | 0 | 0 (0.0%) |
| **Gastrointestinal disorders** | **0** | **0 (0.0%)** | **0** | **0 (0.0%)** | **1** | **1 (1.2%)** | **0** | **0 (0.0%)** |
| Abdominal pain | 0 | 0 (0.0%) | 0 | 0 (0.0%) | 1 | 1 (1.2%) | 0 | 0 (0.0%) |
| **Infections and infestations** | **3** | **2 (2.2%)** | **1** | **1 (1.1%)** | **0** | **0 (0.0%)** | **0** | **0 (0.0%)** |
| Conjunctivitis | 1 | 1 (1.1%) | 0 | 0 (0.0%) | 0 | 0 (0.0%) | 0 | 0 (0.0%) |
| Influenza | 1 | 1 (1.1%) | 0 | 0 (0.0%) | 0 | 0 (0.0%) | 0 | 0 (0.0%) |
| Nasopharyngitis | 0 | 0 (0.0%) | 1 | 1 (1.1%) | 0 | 0 (0.0%) | 0 | 0 (0.0%) |
| Tonsillitis | 1 | 1 (1.1%) | 0 | 0 (0.0%) | 0 | 0 (0.0%) | 0 | 0 (0.0%) |
| **Injury, poisoning and procedural complications** | **1** | **1 (1.1%)** | **0** | **0 (0.0%)** | **0** | **0 (0.0%)** | **0** | **0 (0.0%)** |
| Wound | 1 | 1 (1.1%) | 0 | 0 (0.0%) | 0 | 0 (0.0%) | 0 | 0 (0.0%) |
| **Investigations** | **2** | **1 (1.1%)** | **6** | **2 (2.2%)** | **0** | **0 (0.0%)** | **0** | **0 (0.0%)** |
| Alanine aminotransferase increased | 1 | 1 (1.1%) | 2 | 1 (1.1%) | 0 | 0 (0.0%) | 0 | 0 (0.0%) |
| Aspartate aminotransferase increased | 1 | 1 (1.1%) | 3 | 2 (2.2%) | 0 | 0 (0.0%) | 0 | 0 (0.0%) |
| C-reactive protein increased | 0 | 0 (0.0%) | 1 | 1 (1.1%) | 0 | 0 (0.0%) | 0 | 0 (0.0%) |
| **Reproductive system and breast disorders** | **0** | **0 (0.0%)** | **1** | **1 (1.1%)** | **0** | **0 (0.0%)** | **0** | **0 (0.0%)** |
| Vaginal haemorrhage | 0 | 0 (0.0%) | 1 | 1 (1.1%) | 0 | 0 (0.0%) | 0 | 0 (0.0%) |
|  |  |  |  |  |  |  |  |  |
| During 28 days post any dose | A1 | | A2 | | B1 | | B2 | |
|  | N=93 | | N=91 | | N=90 | | N=93 | |
|  | m | n (%) | m | n (%) | m | n (%) | m | n (%) |
| **Unsolicited AE** | **7** | **5 (5.4%)** | **11** | **6 (6.6%)** | **4** | **3 (3.3%)** | **4** | **3 (3.2%)** |
| **Blood and lymphatic system disorders** | **0** | **0 (0.0%)** | **1** | **1 (1.1%)** | **0** | **0 (0.0%)** | **0** | **0 (0.0%)** |
| Anaemia | 0 | 0 (0.0%) | 1 | 1 (1.1%) | 0 | 0 (0.0%) | 0 | 0 (0.0%) |
| **Gastrointestinal disorders** | **0** | **0 (0.0%)** | **0** | **0 (0.0%)** | **2** | **2 (2.2%)** | **0** | **0 (0.0%)** |
| Abdominal pain | 0 | 0 (0.0%) | 0 | 0 (0.0%) | 1 | 1 (1.1%) | 0 | 0 (0.0%) |
| Abdominal pain upper | 0 | 0 (0.0%) | 0 | 0 (0.0%) | 1 | 1 (1.1%) | 0 | 0 (0.0%) |
| **Infections and infestations** | **3** | **2 (2.2%)** | **1** | **1 (1.1%)** | **1** | **1 (1.1%)** | **2** | **1 (1.1%)** |
| Conjunctivitis | 1 | 1 (1.1%) | 0 | 0 (0.0%) | 0 | 0 (0.0%) | 0 | 0 (0.0%) |
| Influenza | 1 | 1 (1.1%) | 0 | 0 (0.0%) | 0 | 0 (0.0%) | 0 | 0 (0.0%) |
| Nasopharyngitis | 0 | 0 (0.0%) | 1 | 1 (1.1%) | 1 | 1 (1.1%) | 1 | 1 (1.1%) |
| Pharyngitis | 0 | 0 (0.0%) | 0 | 0 (0.0%) | 0 | 0 (0.0%) | 1 | 1 (1.1%) |
| Tonsillitis | 1 | 1 (1.1%) | 0 | 0 (0.0%) | 0 | 0 (0.0%) | 0 | 0 (0.0%) |
| **Injury, poisoning and procedural complications** | **1** | **1 (1.1%)** | **0** | **0 (0.0%)** | **0** | **0 (0.0%)** | **1** | **1 (1.1%)** |
| Joint injury | 0 | 0 (0.0%) | 0 | 0 (0.0%) | 0 | 0 (0.0%) | 1 | 1 (1.1%) |
| Wound | 1 | 1 (1.1%) | 0 | 0 (0.0%) | 0 | 0 (0.0%) | 0 | 0 (0.0%) |
| **Investigations** | **2** | **1 (1.1%)** | **6** | **2 (2.2%)** | **0** | **0 (0.0%)** | **0** | **0 (0.0%)** |
| Alanine aminotransferase increased | 1 | 1 (1.1%) | 2 | 1 (1.1%) | 0 | 0 (0.0%) | 0 | 0 (0.0%) |
| Aspartate aminotransferase increased | 1 | 1 (1.1%) | 3 | 2 (2.2%) | 0 | 0 (0.0%) | 0 | 0 (0.0%) |
| C-reactive protein increased | 0 | 0 (0.0%) | 1 | 1 (1.1%) | 0 | 0 (0.0%) | 0 | 0 (0.0%) |
| **Musculoskeletal and connective tissue disorders** | **0** | **0 (0.0%)** | **0** | **0 (0.0%)** | **0** | **0 (0.0%)** | **1** | **1 (1.1%)** |
| Arthralgia | 0 | 0 (0.0%) | 0 | 0 (0.0%) | 0 | 0 (0.0%) | 1 | 1 (1.1%) |
| **Nervous system disorders** | **0** | **0 (0.0%)** | **1** | **1 (1.1%)** | **0** | **0 (0.0%)** | **0** | **0 (0.0%)** |
| Headache | 0 | 0 (0.0%) | 1 | 1 (1.1%) | 0 | 0 (0.0%) | 0 | 0 (0.0%) |
| **Reproductive system and breast disorders** | **0** | **0 (0.0%)** | **1** | **1 (1.1%)** | **0** | **0 (0.0%)** | **0** | **0 (0.0%)** |
| Vaginal haemorrhage | 0 | 0 (0.0%) | 1 | 1 (1.1%) | 0 | 0 (0.0%) | 0 | 0 (0.0%) |
| **Respiratory, thoracic and mediastinal disorders** | **1** | **1 (1.1%)** | **1** | **1 (1.1%)** | **1** | **1 (1.1%)** | **0** | **0 (0.0%)** |
| Cough | 1 | 1 (1.1%) | 1 | 1 (1.1%) | 1 | 1 (1.1%) | 0 | 0 (0.0%) |

**Supplementary Table 7.** Unsolicited adverse events occurred respectively within 28 days post vaccination with severity by study arms

|  | A1 | A2 | B1 | B2 |
| --- | --- | --- | --- | --- |
|  | N=93 | N=91 | N=90 | N=93 |
| **Post first dose (number of events)** |  |  |  |  |
| **Unsolicited AE** | **1** | **2** | **3** | **4** |
| **Gastrointestinal disorders** | **0** | **0** | **1** | **0** |
| Abdominal pain upper | 0 | 0 | 1 | 0 |
| -Mild | 0 (0.0%) | 0 (0.0%) | 0 (0.0%) | 0 (0.0%) |
| -Moderate | 0 (0.0%) | 0 (0.0%) | 1 (100.0%) | 0 (0.0%) |
| -Severe | 0 (0.0%) | 0 (0.0%) | 0 (0.0%) | 0 (0.0%) |
| **Infections and infestations** | **0** | **0** | **1** | **2** |
| Nasopharyngitis | 0 | 0 | 1 | 1 |
| -Mild | 0 (0.0%) | 0 (0.0%) | 0 (0.0%) | 1 (100.0%) |
| -Moderate | 0 (0.0%) | 0 (0.0%) | 1 (100.0%) | 0 (0.0%) |
| -Severe | 0 (0.0%) | 0 (0.0%) | 0 (0.0%) | 0 (0.0%) |
| Pharyngitis | 0 | 0 | 0 | 1 |
| -Mild | 0 (0.0%) | 0 (0.0%) | 0 (0.0%) | 1 (100.0%) |
| -Moderate | 0 (0.0%) | 0 (0.0%) | 0 (0.0%) | 0 (0.0%) |
| -Severe | 0 (0.0%) | 0 (0.0%) | 0 (0.0%) | 0 (0.0%) |
| **Injury, poisoning and procedural complications** | **0** | **0** | **0** | **1** |
| Joint injury | 0 | 0 | 0 | 1 |
| -Mild | 0 (0.0%) | 0 (0.0%) | 0 (0.0%) | 0 (0.0%) |
| -Moderate | 0 (0.0%) | 0 (0.0%) | 0 (0.0%) | 1 (100.0%) |
| -Severe | 0 (0.0%) | 0 (0.0%) | 0 (0.0%) | 0 (0.0%) |
| **Musculoskeletal and connective tissue disorders** | **0** | **0** | **0** | **1** |
| Arthralgia | 0 | 0 | 0 | 1 |
| -Mild | 0 (0.0%) | 0 (0.0%) | 0 (0.0%) | 1 (100.0%) |
| -Moderate | 0 (0.0%) | 0 (0.0%) | 0 (0.0%) | 0 (0.0%) |
| -Severe | 0 (0.0%) | 0 (0.0%) | 0 (0.0%) | 0 (0.0%) |
| **Nervous system disorders** | **0** | **1** | **0** | **0** |
| Headache | 0 | 1 | 0 | 0 |
| -Mild | 0 (0.0%) | 0 (0.0%) | 0 (0.0%) | 0 (0.0%) |
| -Moderate | 0 (0.0%) | 1 (100.0%) | 0 (0.0%) | 0 (0.0%) |
| -Severe | 0 (0.0%) | 0 (0.0%) | 0 (0.0%) | 0 (0.0%) |
| **Respiratory, thoracic and mediastinal disorders** | **1** | **1** | **1** | **0** |
| Cough | 1 | 1 | 1 | 0 |
| -Mild | 0 (0.0%) | 1 (100.0%) | 1 (100.0%) | 0 (0.0%) |
| -Moderate | 1 (100.0%) | 0 (0.0%) | 0 (0.0%) | 0 (0.0%) |
| -Severe | 0 (0.0%) | 0 (0.0%) | 0 (0.0%) | 0 (0.0%) |

|  | A1 | A2 | B1 | B2 |
| --- | --- | --- | --- | --- |
|  | N=89 | N=90. | N=84 | N=86 |
| **Post second dose (number of events)** |  |  |  |  |
| **Unsolicited AE** | 6 | 9 | 1 | 0 |
| **Blood and lymphatic system disorders** | **0** | **1** | **0** | **0** |
| Anaemia | 0 | 1 | 0 | 0 |
| -Mild | 0 (0.0%) | 0 (0.0%) | 0 (0.0%) | 0 (0.0%) |
| -Moderate | 0 (0.0%) | 1 (100.0%) | 0 (0.0%) | 0 (0.0%) |
| -Severe | 0 (0.0%) | 0 (0.0%) | 0 (0.0%) | 0 (0.0%) |
| **Gastrointestinal disorders** | **0** | **0** | **1** | **0** |
| Abdominal pain | 0 | 0 | 1 | 0 |
| -Mild | 0 (0.0%) | 0 (0.0%) | 1 (100.0%) | 0 (0.0%) |
| -Moderate | 0 (0.0%) | 0 (0.0%) | 0 (0.0%) | 0 (0.0%) |
| -Severe | 0 (0.0%) | 0 (0.0%) | 0 (0.0%) | 0 (0.0%) |
| **Infections and infestations** | **3** | **1** | **0** | **0** |
| Conjunctivitis | 1 | 0 | 0 | 0 |
| -Mild | 1 (100.0%) | 0 (0.0%) | 0 (0.0%) | 0 (0.0%) |
| -Moderate | 0 (0.0%) | 0 (0.0%) | 0 (0.0%) | 0 (0.0%) |
| -Severe | 0 (0.0%) | 0 (0.0%) | 0 (0.0%) | 0 (0.0%) |
| Influenza | 1 | 0 | 0 | 0 |
| -Mild | 1 (100.0%) | 0 (0.0%) | 0 (0.0%) | 0 (0.0%) |
| -Moderate | 0 (0.0%) | 0 (0.0%) | 0 (0.0%) | 0 (0.0%) |
| -Severe | 0 (0.0%) | 0 (0.0%) | 0 (0.0%) | 0 (0.0%) |
| Nasopharyngitis | 0 | 1 | 0 | 0 |
| -Mild | 0 (0.0%) | 1 (100.0%) | 0 (0.0%) | 0 (0.0%) |
| -Moderate | 0 (0.0%) | 0 (0.0%) | 0 (0.0%) | 0 (0.0%) |
| -Severe | 0 (0.0%) | 0 (0.0%) | 0 (0.0%) | 0 (0.0%) |
| Tonsillitis | 1 | 0 | 0 | 0 |
| -Mild | 1 (100.0%) | 0 (0.0%) | 0 (0.0%) | 0 (0.0%) |
| -Moderate | 0 (0.0%) | 0 (0.0%) | 0 (0.0%) | 0 (0.0%) |
| -Severe | 0 (0.0%) | 0 (0.0%) | 0 (0.0%) | 0 (0.0%) |
| **Injury, poisoning and procedural complications** | **1** | **0** | **0** | **0** |
| Wound | 1 | 0 | 0 | 0 |
| -Mild | 0 (0.0%) | 0 (0.0%) | 0 (0.0%) | 0 (0.0%) |
| -Moderate | 1 (100.0%) | 0 (0.0%) | 0 (0.0%) | 0 (0.0%) |
| -Severe | 0 (0.0%) | 0 (0.0%) | 0 (0.0%) | 0 (0.0%) |
| **Investigations** | **2** | **6** | **0** | **0** |
| Alanine aminotransferase increased | 1 | 2 | 0 | 0 |
| -Mild | 1 (100.0%) | 0 (0.0%) | 0 (0.0%) | 0 (0.0%) |
| -Moderate | 0 (0.0%) | 1 (50.0%) | 0 (0.0%) | 0 (0.0%) |
| -Severe | 0 (0.0%) | 1 (50.0%) | 0 (0.0%) | 0 (0.0%) |
| Aspartate aminotransferase increased | 1 | 3 | 0 | 0 |
| -Mild | 1 (100.0%) | 1 (33.3%) | 0 (0.0%) | 0 (0.0%) |
| -Moderate | 0 (0.0%) | 1 (33.3%) | 0 (0.0%) | 0 (0.0%) |
| -Severe | 0 (0.0%) | 1 (33.3%) | 0 (0.0%) | 0 (0.0%) |
| C-reactive protein increased | 0 | 1 | 0 | 0 |
| -Mild | 0 (0.0%) | 0 (0.0%) | 0 (0.0%) | 0 (0.0%) |
| -Moderate | 0 (0.0%) | 1 (100.0%) | 0 (0.0%) | 0 (0.0%) |
| -Severe | 0 (0.0%) | 0 (0.0%) | 0 (0.0%) | 0 (0.0%) |
| **Reproductive system and breast disorders** | **0** | **1** | **0** | **0** |
| Vaginal haemorrhage | 0 | 1 | 0 | 0 |
| -Mild | 0 (0.0%) | 1 (100.0%) | 0 (0.0%) | 0 (0.0%) |
| -Moderate | 0 (0.0%) | 0 (0.0%) | 0 (0.0%) | 0 (0.0%) |
| -Severe | 0 (0.0%) | 0 (0.0%) | 0 (0.0%) | 0 (0.0%) |

|  | A1 | A2 | B1 | B2 |
| --- | --- | --- | --- | --- |
|  | N=93 | N=91 | N=90 | N=93 |
| **Post any dose (number of events)** |  |  |  |  |
| **Unsolicited AE** | 7 | 11 | 4 | 4 |
| **Blood and lymphatic system disorders** | 0 | 1 | 0 | 0 |
| Anaemia | 0 | 1 | 0 | 0 |
| -Mild | 0 (0.0%) | 0 (0.0%) | 0 (0.0%) | 0 (0.0%) |
| -Moderate | 0 (0.0%) | 1 (100.0%) | 0 (0.0%) | 0 (0.0%) |
| -Severe | 0 (0.0%) | 0 (0.0%) | 0 (0.0%) | 0 (0.0%) |
| **Gastrointestinal disorders** | 0 | 0 | 2 | 0 |
| Abdominal pain | 0 | 0 | 1 | 0 |
| -Mild | 0 (0.0%) | 0 (0.0%) | 1 (100.0%) | 0 (0.0%) |
| -Moderate | 0 (0.0%) | 0 (0.0%) | 0 (0.0%) | 0 (0.0%) |
| -Severe | 0 (0.0%) | 0 (0.0%) | 0 (0.0%) | 0 (0.0%) |
| Abdominal pain upper | 0 | 0 | 1 | 0 |
| -Mild | 0 (0.0%) | 0 (0.0%) | 0 (0.0%) | 0 (0.0%) |
| -Moderate | 0 (0.0%) | 0 (0.0%) | 1 (100.0%) | 0 (0.0%) |
| -Severe | 0 (0.0%) | 0 (0.0%) | 0 (0.0%) | 0 (0.0%) |
| **Infections and infestations** | 3 | 1 | 1 | 2 |
| Conjunctivitis | 1 | 0 | 0 | 0 |
| -Mild | 1 (100.0%) | 0 (0.0%) | 0 (0.0%) | 0 (0.0%) |
| -Moderate | 0 (0.0%) | 0 (0.0%) | 0 (0.0%) | 0 (0.0%) |
| -Severe | 0 (0.0%) | 0 (0.0%) | 0 (0.0%) | 0 (0.0%) |
| Influenza | 1 | 0 | 0 | 0 |
| -Mild | 1 (100.0%) | 0 (0.0%) | 0 (0.0%) | 0 (0.0%) |
| -Moderate | 0 (0.0%) | 0 (0.0%) | 0 (0.0%) | 0 (0.0%) |
| -Severe | 0 (0.0%) | 0 (0.0%) | 0 (0.0%) | 0 (0.0%) |
| Nasopharyngitis | 0 | 1 | 1 | 1 |
| -Mild | 0 (0.0%) | 1 (100.0%) | 0 (0.0%) | 1 (100.0%) |
| -Moderate | 0 (0.0%) | 0 (0.0%) | 1 (100.0%) | 0 (0.0%) |
| -Severe | 0 (0.0%) | 0 (0.0%) | 0 (0.0%) | 0 (0.0%) |
| Pharyngitis | 0 | 0 | 0 | 1 |
| -Mild | 0 (0.0%) | 0 (0.0%) | 0 (0.0%) | 1 (100.0%) |
| -Moderate | 0 (0.0%) | 0 (0.0%) | 0 (0.0%) | 0 (0.0%) |
| -Severe | 0 (0.0%) | 0 (0.0%) | 0 (0.0%) | 0 (0.0%) |
| Tonsillitis | 1 | 0 | 0 | 0 |
| -Mild | 1 (100.0%) | 0 (0.0%) | 0 (0.0%) | 0 (0.0%) |
| -Moderate | 0 (0.0%) | 0 (0.0%) | 0 (0.0%) | 0 (0.0%) |
| -Severe | 0 (0.0%) | 0 (0.0%) | 0 (0.0%) | 0 (0.0%) |
| **Injury, poisoning and procedural complications** | 1 | 0 | 0 | 1 |
| Joint injury | 0 | 0 | 0 | 1 |
| -Mild | 0 (0.0%) | 0 (0.0%) | 0 (0.0%) | 0 (0.0%) |
| -Moderate | 0 (0.0%) | 0 (0.0%) | 0 (0.0%) | 1 (100.0%) |
| -Severe | 0 (0.0%) | 0 (0.0%) | 0 (0.0%) | 0 (0.0%) |
| Wound | 1 | 0 | 0 | 0 |
| -Mild | 0 (0.0%) | 0 (0.0%) | 0 (0.0%) | 0 (0.0%) |
| -Moderate | 1 (100.0%) | 0 (0.0%) | 0 (0.0%) | 0 (0.0%) |
| -Severe | 0 (0.0%) | 0 (0.0%) | 0 (0.0%) | 0 (0.0%) |
| **Investigations** | 2 | 6 | 0 | 0 |
| Alanine aminotransferase increased | 1 | 2 | 0 | 0 |
| -Mild | 1 (100.0%) | 0 (0.0%) | 0 (0.0%) | 0 (0.0%) |
| -Moderate | 0 (0.0%) | 1 (50.0%) | 0 (0.0%) | 0 (0.0%) |
| -Severe | 0 (0.0%) | 1 (50.0%) | 0 (0.0%) | 0 (0.0%) |
| Aspartate aminotransferase increased | 1 | 3 | 0 | 0 |
| -Mild | 1 (100.0%) | 1 (33.3%) | 0 (0.0%) | 0 (0.0%) |
| -Moderate | 0 (0.0%) | 1 (33.3%) | 0 (0.0%) | 0 (0.0%) |
| -Severe | 0 (0.0%) | 1 (33.3%) | 0 (0.0%) | 0 (0.0%) |
| C-reactive protein increased | 0 | 1 | 0 | 0 |
| -Mild | 0 (0.0%) | 0 (0.0%) | 0 (0.0%) | 0 (0.0%) |
| -Moderate | 0 (0.0%) | 1 (100.0%) | 0 (0.0%) | 0 (0.0%) |
| -Severe | 0 (0.0%) | 0 (0.0%) | 0 (0.0%) | 0 (0.0%) |
| **Musculoskeletal and connective tissue disorders** | 0 | 0 | 0 | 1 |
| Arthralgia | 0 | 0 | 0 | 1 |
| -Mild | 0 (0.0%) | 0 (0.0%) | 0 (0.0%) | 1 (100.0%) |
| -Moderate | 0 (0.0%) | 0 (0.0%) | 0 (0.0%) | 0 (0.0%) |
| -Severe | 0 (0.0%) | 0 (0.0%) | 0 (0.0%) | 0 (0.0%) |
| **Nervous system disorders** | 0 | 1 | 0 | 0 |
| Headache | 0 | 1 | 0 | 0 |
| -Mild | 0 (0.0%) | 0 (0.0%) | 0 (0.0%) | 0 (0.0%) |
| -Moderate | 0 (0.0%) | 1 (100.0%) | 0 (0.0%) | 0 (0.0%) |
| -Severe | 0 (0.0%) | 0 (0.0%) | 0 (0.0%) | 0 (0.0%) |
| **Reproductive system and breast disorders** | 0 | 1 | 0 | 0 |
| Vaginal haemorrhage | 0 | 1 | 0 | 0 |
| -Mild | 0 (0.0%) | 1 (100.0%) | 0 (0.0%) | 0 (0.0%) |
| -Moderate | 0 (0.0%) | 0 (0.0%) | 0 (0.0%) | 0 (0.0%) |
| -Severe | 0 (0.0%) | 0 (0.0%) | 0 (0.0%) | 0 (0.0%) |
| **Respiratory, thoracic and mediastinal disorders** | 1 | 1 | 1 | 0 |
| Cough | 1 | 1 | 1 | 0 |
| -Mild | 0 (0.0%) | 1 (100.0%) | 1 (100.0%) | 0 (0.0%) |
| -Moderate | 1 (100.0%) | 0 (0.0%) | 0 (0.0%) | 0 (0.0%) |
| -Severe | 0 (0.0%) | 0 (0.0%) | 0 (0.0%) | 0 (0.0%) |
